# Complexity-Building ESIPT-Assisted Synthesis of Fused Polyheterocyclic Sulfonamides

**DOI:** 10.3390/molecules28186549

**Published:** 2023-09-10

**Authors:** Srinivas Beduru, Andrei G. Kutateladze

**Affiliations:** Department of Chemistry and Biochemistry, University of Denver, Denver, CO 80208, USA; srinivas.beduru@du.edu

**Keywords:** photoassisted synthetic chemistry, ESIPT, polyheterocycles, sulfonamides, molecular complexity

## Abstract

Excited State Intramolecular Proton Transfer (ESIPT), originally discovered and explored in depth in a number of extensive photophysical studies, is more recently rediscovered as a powerful synthetic tool, offering rapid access to complex polyheterocycles. In our prior work we have employed ESIPT in aromatic *o*-keto amines and amides, leading to diverse primary photoproducts—complex quinolinols or azacanes possessing a fused lactam moiety—which could additionally be modified in short, high-yielding postphotochemical reactions to further grow complexity of the heterocyclic core scaffold and/or to decorate it with additional functional groups. Given that sulfonamides are generally known as privileged substructures, in this study we pursued two goals: (i) To explore whether sulfonamides could behave as proton donors in the context of ESIPT-initiated photoinduced reactions; (ii) To assess the scope of subsequent complexity-building photochemical and postphotochemical steps, which give access to polyheterocyclic molecular cores with fused cyclic sulfonamide moieties. In this work we show that this is indeed the case. Simple sulfonamide-containing photoprecursors produced the sought-after heterocyclic products in experimentally simple photochemical reactions accompanied by significant step-normalized complexity increases as corroborated by the Böttcher complexity scores.

## 1. Introduction

Excited State Intramolecular Proton Transfer (ESIPT) in aromatic *o*-amido-ketones was studied extensively by photophysicists, including the groundbreaking work of Smith and Barbara [1] and later Blank [2]. Relatively recently it was rediscovered as a useful synthetic tool, offering rapid access to complex polyheterocycles [3,4,5], especially in the context of diversity-oriented synthesis [6]. Porco and Stephenson employed ESIPT in 3-hydroxyflavone derivatives and methyl cinnamate [7], *trans*-stilbene [8], and *trans*,*trans*-1,4-diphenyl-1,3-butadiene (DPBD) [9] as dipolarophiles generating 1,3-dipolar reactive intermediates and accessing [3+2] photocycloaddition products [10].

**Scheme 1:**
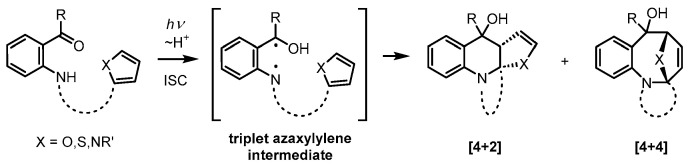
Synthetic utilization of ESIPT-generated azaxylylenes.

We have demonstrated that ESIPT in aromatic *o*-amino ketones and their derivatives generates aza-*o*-xylylenes, capable of intramolecular cycloadditions to tethered unsaturated pendants, including five-membered aromatic heterocycles (see Scheme 1) and benzenoid aromatic moieties [3,4,5]. While the *ortho*-amido and amino ketones were explored most extensively, we also achieved similar synthetic goals with *ortho*-hydroxy ketones accessing complex 2,6-epoxybenzazocines [11]. *ESIPT in sulfonamides, however, was not studied*.

Given that the sulfonamide moiety is prominently featured in approved drugs and other biologically active compounds [12,13,14,15,16,17,18,19], in this study we set out to explore ESIPT in sulfonamides and assess its synthetic utility. As noted above, another aspect of this work was to evaluate the compatibility of this molecular complexity-building photoassisted synthetic chemistry approach with fundamental principles of combinatorial chemistry and diversity-oriented synthesis [6]. This includes the mutually interrelated goals of: (i) Modular design of photoprecursors from readily available building blocks (ii) Incursions into the unexplored areas of chemical space, especially via diversification of the core polyheterocyclic scaffolds.

## 2. Results

**Scheme 2:**
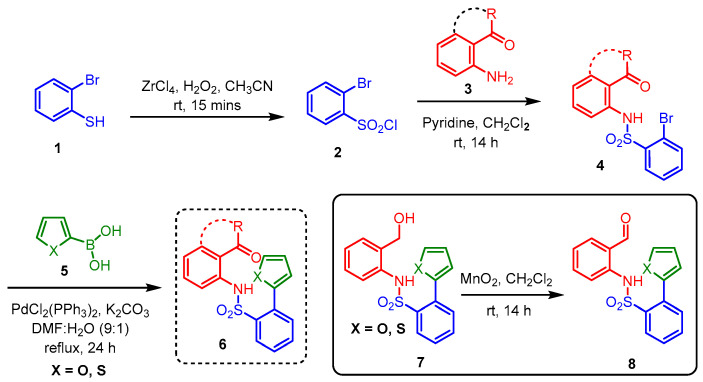
Modular synthesis of photoprecursors.

Photoprecursor sulfonamides were readily synthesized in a modular fashion as shown in Scheme 2. Well-established high yielding reactions were chosen to link the building blocks together, i.e., the likes of Suzuki coupling or effective coupling of aromatic amines with sulfonyl chlorides. Generally, three diversity inputs (the photoactive core, i.e., aminoketone, the sulfonamide tether, and the unsaturated pendant) allow for added flexibility in exploring the chemical space traversed by the resulting photoproducts. Subsequent postphotochemical modifications offer opportunities for additional diversity inputs.

Irradiation of photoprecursors in DMSO with 365 nm UV LED yielded the products of [4+2] or [4+4] cycloadditions in good yields (see Table 1).

The now-ubiquitous UV LEDs @365 nm, based on Al-doped gallium nitride, with rather high radiant power are available commercially, making them the UV source of choice for large scale irradiations. LED irradiators with shorter wavelengths are becoming more accessible but their prices are still significantly higher. Additionally, the higher energy photons are more prone to causing unwanted side reactions. The 365 nm wavelength offers the optimal cost-benefit outcomes, provided that the photoprecursors absorb at this wavelength. As shown in Table 2, the precursor sulfonamides do have significant absorption at 365 nm, with molar extinction coefficients ranging from 830 to 1770 M^−1^ cm^−1^.

**Scheme 3:**
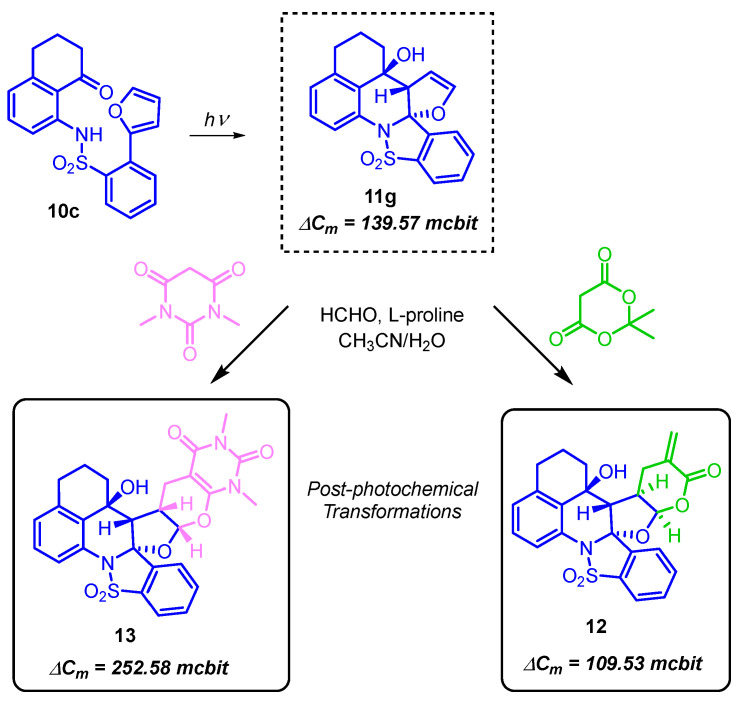
Postphotochemical modifications of primary photoproduct **11g** resulting in complex polyheterocycles **12** and **13** (Böttcher complexity scores are shown).

Postphotochemical modifications of **12** and **13** were carried out as shown in Scheme 3 via the [4+2] (hetero-Diels-Alder) reaction of the dihydrofuran moiety in primary photoproduct (**11g**) and hetero-dienes generated in situ from either Meldrum’s acid (green) or *N*,*N*-dimethylbarbiturate (pink) and formaldehyde, under L-proline catalysis.

## 3. Discussion

**Scheme 4:**
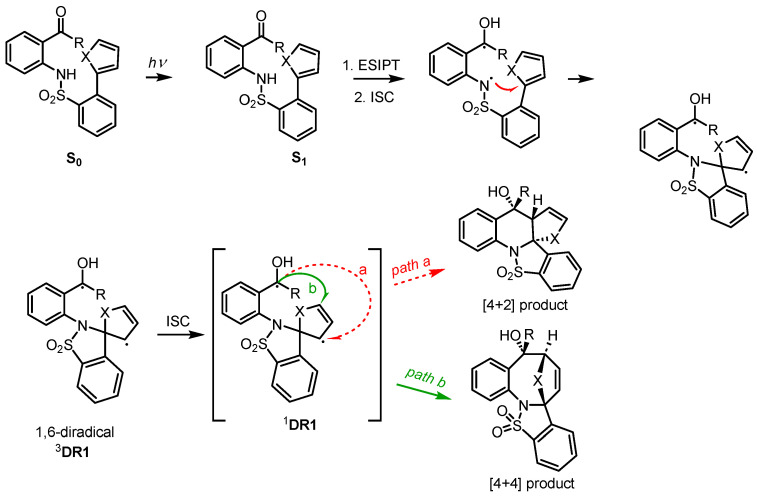
Plausible mechanistic rationale.

The results presented above indicate clearly that sulfonamides undergo ESIPT, generating N-sulfonyl aza-*o*-xylylenes capable of intramolecular cycloadditions to yield complex heterocycles possessing a fused cyclic sulfonamide moiety. Detailed photophysical study is ongoing and will be published in due course. Given that both [4+2] and [4+4] reaction topologies are realized, we hypothesize that—as in the previously reported reactions of amides—sulfonamides most likely react in their triplet state. This is a beneficial feature, as it expands the synthetic utility of these reactions giving access to higher diversity of polyheterocyclic cores in the primary photoproducts, i.e., sulfonylated quinolinols and sulfonylated azacanes. Overall, the yields of sulfonamides are generally higher than lactams derived from amidoketones. This could be attributed either to more efficient ESIPT in sulfonamides, or the higher reactivity of *N*-sulfonyl aza-*o*-xylylenes (or both). A proposed mechanistic rationale is presented in Scheme 4.

The regiochemical outcome, i.e., the competition between *path a* and *path b* in Scheme 4, is likely being controlled by the spin density in the 1,6-diradical **DR1** formed as a result of the initial attack of the *N*-centered radical on the π-system of the hetero-dienic (i.e., furan or thiophene) pendant. In this study, photoprecursors containing thiophene produced the [4+2]-product exclusively (*path a*), while the reactions with furan gave both [4+2] and [4+4] products, indicating that *path b* was becoming increasingly more competitive with *path a* in the case of furan pendants.

Product structures were determined by NMR. Given the complexity of these polyheterocyclic products, experimental NMR characterization was augmented with computational study, i.e., the experimental data were matched with spectra calculated with our machine learning-augmented DFT method, DU8ML [20,21]. All structures showed convincing matches with the computed data—for example, rmsd values for ^13^C NMR chemical shifts were in the 1.1–1.3 ppm range and the calculated spin–spin coupling values were in keeping with the experimental observations. A typical result of DU8ML calculations of NMR parameters is shown in Table 3.

## 4. Materials and Methods

### 4.1. General Information

Common solvents were purchased from Fisher Scientific (Waltham, MA, USA) and used as is. Common reagents, 2-keto amines, boronic acids, and thiols, were purchased from Sigma-Aldrich (St. Louis, MO, USA), TCI America, AK Scientific, Oakwood Chemical or AstaTech and used without additional purification. NMR spectra were recorded at 25 °C on a Bruker Avance III 500 MHz instrument (Billerica, MA, USA) in CDCl_3_ (unless noted otherwise) using residual solvent peaks as an internal standard (*δ*H 7.26 ppm, *δ*C 77.16 ppm for CDCl_3_; *δ*H 2.50 ppm, *δ*C 39.52 ppm for DMSO-*d*_6_). The description of signals includes s = singlet, d = doublet, dd = doublet of doublets, t = triplet, dt = doublet of triplets, td = triplet of doublets, q = quartet, m = multiplet, br.s = broad singlet. The structural assignments were made with additional information from gCOSY, gHSQC, and gHMBC experiments. Flash column chromatography was performed using Teledyne Ultra-Pure Silica Gel (230–400 mesh) on a Teledyne Isco Combiflash Rf (Lincoln, NE, USA). The light-promoted reactions were carried out using in-house built UV LED-based illuminators equipped with seven (total power 20.3 W) LED Engin chips (18 V, 700 mA, 2.9 W, λ = 365 nm). Borosilicate glass reaction vessels were typically distanced from a light source in a range of 5–7 cm.

### 4.2. Experimental Procedures and Characterization Data

#### 4.2.1. General Procedure for Sulfonamide Reactions (General Procedure A)

A mixture of 2-bromobenzenethiol (1.0 g; 5.3 mmol), 30% H_2_O_2_ (1.5 mL, 8.0 mmol), and ZrCl_4_ (1.23 g, 5.3 mmol) were stirred in MeCN (15 mL) at 25 °C for 10 min. The reaction mixture was quenched by adding H_2_O (30 mL), and extracted with EtOAc (4 × 50 mL). The organic extract was dried with anhydrous Na_2_SO_4_ and the filtrate was evaporated under vacuum to afford **2** as red crystals (1.23 g, 92%).

To a stirred solution of 2-bromobenzenesulfonyl chloride (**2**, 3.91 mmol), the corresponding aniline (3.91 mmol), and anhydrous pyridine (1.0 mL, 11.7 mmol) in anhydrous dichloromethane (15 mL) was stirred under an argon atmosphere. The resulting mixture was stirred at room temperature for 14 h. After completion of the reaction (the progress of the reaction was monitored by Thin layer chromatography), the reaction mixture was diluted with washed with saturated NaHCO_3_ (20 mL). The reaction mixture was extracted with CH_2_Cl_2_ (3 × 30 mL). The combined organic extracts were washed with water (2 × 40 mL), separated, dried over anhydrous Na_2_SO_4_, then concentrated in vacuo to give a crude yellow oil. The crude product was purified by flash chromatography on silica gel.

#### 4.2.2. General Procedure for Synthesis of Aldehyde Photoprecursors (General Procedure B)

The bromide (2.0 mmol, 1.0 equiv.), the corresponding boronic acid (3.0 mmol, 1.5 equiv.), PdCl_2_(PPh_3_)_2_ (0.07 mmol, 0.04 equiv.), and K_2_CO_3_ (8.0 mmol, 4.0 equiv.) are suspended in DMF:H_2_O (9:1) and heated to reflux in oil bath for 14 h. Upon completion of the reaction as indicated by ^1^H NMR, the mixture was diluted with EtOAc (30 mL) and washed with a saturated solution of NaHCO_3_ (2 × 40 mL). Combined organic phase was washed with H_2_O (20 mL) and dried over anhydrous Na_2_SO_4_. After concentration, a crude product was used for the next oxidation reaction. To a stirred solution of the corresponding alcohol (2.5 mmol, 1.0 equiv.) in CH_2_Cl_2_ (20 mL), was added MnO_2_ (12.5 mmol; 5.0 equiv.) at ambient temperature, and the resulting mixture was stirred at room temperature overnight. Upon completion (the progress of the reaction was monitored by ^1^H NMR), the solids were filtered off by passing the mixture through celite pad, and the pad was washed with additional 20 mL of CH_2_Cl_2_. After concentration, crude product was purified by flash chromatography on silica gel to give a desired photoprecursor.

*N-(2-Formylphenyl)-2-(furan-2-yl)benzenesulfonamide* (**8a**). Following the general procedure **B**, compound **8a** (126 mg, 79%) was obtained from the oxidation of **7a** (160 mg) as a brown solid. ^1^H NMR (500 MHz, CDCl_3_): *δ* 11.08 (s, 1H), 9.81 (s, 1H), 8.32 (d, *J* = 8.0 Hz, 1H), 7.70 (d, *J* = 1.8 Hz, 1H), 7.60 (ddd, *J* = 8.7, 6.1, 4.3 Hz, 4H), 7.54–7.48 (m, 1H), 7.45 (t, *J* = 8.2 Hz, 1H), 7.12 (t, *J* = 7.5 Hz, 1H), 6.94 (d, *J* = 3.4 Hz, 1H), 6.59 (dd, *J* = 3.3, 1.8 Hz, 1H) ppm; ^13^C NMR (126 MHz, CDCl_3_): *δ* 194.11, 149.86, 143.75, 139.69, 136.08, 136.03, 135.65, 133.05, 131.24, 130.98, 130.09, 127.96, 122.24, 121.42, 116.29, 111.88, 111.82 ppm; HRMS (ESI) *m*/*z*: [M + Na]^+^ calcd for C_17_H_13_NO_4_SNa 350.0457; found: 350.0453.

*N-(2-Formylphenyl)-2-(thiophen-2-yl)benzenesulfonamide* (**8b**). Following the general procedure **B**, compound **8b** (190 mg, 83%) was obtained from the oxidation of **7b** (230 mg) as a colorless red powder. ^1^H NMR (500 MHz, CDCl_3_): *δ* 10.32 (s, 1H), 9.66 (s, 1H), 8.42–8.36 (m, 1H), 7.56 (ddt, *J* = 10.2, 4.9, 2.0 Hz, 3H), 7.43 (d, *J* = 4.0 Hz, 1H), 7.41–7.33 (m, 2H), 7.28 (s, 1H), 7.27 (s, 1H), 7.16 (dd, *J* = 5.2, 3.5 Hz, 1H), 7.09 (t, *J* = 7.5 Hz, 1H). ppm; ^13^C NMR (126 MHz, CDCl_3_): *δ* 193.66, 139.35, 137.96, 137.76, 135.74, 135.45, 134.07, 133.72, 132.83, 130.83, 129.90, 128.22, 127.64, 127.12, 122.06, 121.11, 115.69 ppm; HRMS (ESI) *m*/*z*: [M + H]^+^ calcd for C_17_H_14_NO_3_S_2_ 344.0409; found: 344.0416.

*N-(2-Formylphenyl)-2-(furan-2-yl)-4,5-dimethoxybenzenesulfonamide* (**8c**). Following the general procedure **B**, compound **8c** (130 mg, 73%) was obtained from the oxidation of **7c** (180 mg) as a white solid. ^1^H NMR (500 MHz, CDCl_3_): *δ* 10.90 (s, 1H), 9.77 (s, 1H), 7.79 (s, 1H), 7.58 (d, *J* = 8.4 Hz, 2H), 7.49 (d, *J* = 8.3 Hz, 1H), 7.43 (t, *J* = 7.9 Hz, 1H), 7.12 (t, *J* = 7.5 Hz, 1H), 7.01 (s, 1H), 6.92 (d, *J* = 3.4 Hz, 1H), 6.57 (dd, *J* = 3.5, 1.9 Hz, 1H), 4.02 (s, 3H), 3.93 (s, 3H) ppm; ^13^C NMR (126 MHz, CDCl_3_): 194.01, 152.12, 149.39, 148.05, 143.18, 139.70, 136.01, 135.55, 128.07, 124.08, 122.20, 121.36, 116.28, 113.65, 113.37, 111.84, 111.52, 56.51, 56.24 ppm; HRMS (ESI) *m*/*z*: [M + H]^+^ calcd for C_19_H_18_NO_6_S 388.0849; found: 388.0873.

*N-(2-Formylphenyl)-4,5-dimethoxy-2-(thiophen-2-yl)benzenesulfonamide* (**8d**). Following the general procedure **B**, compound **8d** (135 mg, 91%) was obtained from the oxidation of **7d** (150 mg) as yellow solid. ^1^H NMR (500 MHz, CDCl_3_): *δ* 10.26 (s, 1H), 9.65 (s, 1H), 7.84 (s, 1H), 7.55 (d, *J* = 7.9 Hz, 1H), 7.42–7.32 (m, 3H), 7.25 (d, *J* = 8.5 Hz, 1H), 7.14–7.05 (m, 2H), 6.82 (s, 1H), 4.05 (s, 3H), 3.92–3.84 (m, 3H) ppm; ^13^C NMR (126 MHz, CDCl_3_): 193.63, 151.79, 148.26, 139.47, 137.82, 135.70, 135.43, 129.68, 127.54, 127.33, 126.75, 122.59, 121.94, 121.11, 116.04, 115.68, 113.29, 56.60, 56.25 ppm; HRMS (ESI) *m*/*z*: [M + Na]^+^ calcd for C_19_H_17_NO_5_S_2_Na 426.0440; found: 426.0441.

#### 4.2.3. General Procedure for Synthesis of Keto Photoprecursors (General Procedure C)

The bromide (2.0 mmol, 1.0 equiv.), the corresponding boronic acid (3.0 mmol, 1.5 equiv.), PdCl_2_(PPh_3_)_2_ (0.07 mmol, 0.04 equiv.), and K_2_CO_3_ (8.0 mmol, 4.0 equiv.) are suspended in DMF:H_2_O (9:1) and heated to reflux in oil bath for 14 h. Upon completion of the reaction as indicated by ^1^H NMR, the mixture was diluted with EtOAc (30 mL) and washed with a saturated solution of NaHCO_3_ (2 × 40 mL). Combined organic phase was washed with H_2_O (20 mL) and dried over anhydrous Na_2_SO_4_. After concentration, the crude product was purified by flash chromatography on silica gel to give a desired keto photoprecursor.

*2-(Furan-2-yl)-N-(8-oxo-5,6,7,8-tetrahydronaphthalen-1-yl)benzenesulfonamide* (**10c**). Following the general procedure **C**, compound **10c** (184 mg, 64%) was obtained from the suzuki coupling of **9b** with **6a** (300 mg) as white solid. ^1^H NMR (500 MHz, CDCl_3_): *δ* 12.16 (s, 1H), 8.31 (d, *J* = 8.0 Hz, 1H), 7.67–7.53 (m, 3H), 7.48 (t, *J* = 7.7 Hz, 1H), 7.33 (d, *J* = 8.4 Hz, 1H), 7.24 (t, *J* = 8.0 Hz, 1H), 6.98 (d, *J* = 3.4 Hz, 1H), 6.80 (d, *J* = 7.4 Hz, 1H), 6.60–6.55 (m, 1H), 2.90 (t, *J* = 6.1 Hz, 2H), 2.62 (t, *J* = 6.4 Hz, 2H), 2.04 (p, *J* = 6.3 Hz, 2H) ppm; ^13^C NMR (126 MHz, CDCl_3_): *δ* 201.78, 149.73, 146.35, 143.42, 140.74, 136.42, 134.46, 132.71, 131.06, 130.97, 130.07, 127.82, 122.30, 118.01, 114.63, 112.02, 111.86, 40.17, 30.76, 22.55 ppm; HRMS (ESI) *m*/*z*: [M + Na]^+^ calcd for C_20_H_17_NO_4_SNa 390.0770; found: 390.0758.

#### 4.2.4. Irradiation of Photo Precursors (General Procedure D)

A solution of photo precursor (0.30 mmol) in DMSO (80 mL, unless otherwise mentioned) was degassed by bubbling of nitrogen or argon for 45 min. The solution was irradiated with UV LED-based illuminator, seven 2.9 W (total power 20.3 W) @ 365 nm LED Engin chips. After completion of the reaction (progress of the reaction was monitored by ^1^H NMR), the solvent was removed under vacuum, and a residue was subjected to purification by flash chromatography on silica gel to obtain photoproducts with moderate to good yields.

*(5S,6S,8aS)-5-Hydroxy-5,6-dihydro-6,8a-epoxybenzo[g]benzo[4,5]isothiazolo[2,3-a]azocine 13,13-dioxide* (**11a**). General procedure **D** was followed on 0.100 g (0.30 mmol) scale of photoprecursor **8a**. After the photochemical reaction (irradiation time = 1 h), the crude product was purified by flash chromatography (SiO_2_, 0–40% ethyl acetate in hexanes) which afforded 87 mg (87%) of photoproduct (**11a**) as a white amorphous solid. ^1^H NMR (500 MHz, DMSO-*d*_6_) *δ* 8.08 (d, *J* = 7.6 Hz, 1H), 7.91–7.86 (m, 1H), 7.85–7.81 (m, 2H), 7.73 (dd, *J* = 5.9, 3.4 Hz, 1H), 7.65 (d, *J* = 7.4 Hz, 1H), 7.35 (dd, *J* = 6.1, 3.4 Hz, 2H), 6.79 (dd, *J* = 5.7, 1.8 Hz, 1H), 6.30 (d, *J* = 6.5 Hz, 1H), 6.12 (d, *J* = 5.7 Hz, 1H), 5.00 (s, 1H), 4.97 (dd, *J* = 6.5, 3.3 Hz, 1H) ppm; ^13^C NMR (126 MHz, DMSO-*d*_6_) *δ* 138.95, 137.44, 135.61, 135.13, 134.64, 132.30, 130.41, 129.03, 128.08, 126.95, 126.77, 126.26, 125.51, 121.37, 99.35, 84.95, 74.51 ppm; HRMS (ESI) *m*/*z*: [M + Na]^+^ calcd for C_18_H_15_NO_4_SNa 350.0457; found: 350.0455.

*(4bR,7aS,8R)-8-Hydroxy-7a,8-dihydrobenzo[4,5]isothiazolo[2,3-a]thieno[2,3-b]quinoline 14,14-dioxide* (**11b**). General procedure **D** was followed on 0.100 g (0.29 mmol) scale of photoprecursor **8b**. After the photochemical reaction (irradiation time = 4 h), the crude product was purified by flash chromatography (SiO_2_, 0–60% ethyl acetate in hexanes) which afforded 81 mg (81%) of photoproduct (**11b**) as a brown solid. ^1^H NMR (500 MHz, CDCl_3_) *δ* 7.85 (dtd, *J* = 23.1, 16.9, 15.1, 7.9 Hz, 3H), 7.64 (s, 1H), 7.64–7.49 (m, 2H), 7.42 (q, *J* = 4.6 Hz, 2H), 6.24 (dd, *J* = 6.7, 2.7 Hz, 1H), 5.41 (dd, *J* = 6.7, 2.3 Hz, 1H), 5.05 (d, *J* = 5.7 Hz, 1H), 4.45 (dt, *J* = 5.7, 2.6 Hz, 1H), 2.40 (s, 1H; OH) ppm; ^13^C NMR (126 MHz, CDCl_3_) *δ* 140.12, 136.14, 134.27, 133.88, 130.91, 130.27, 128.28, 128.03, 127.28, 126.06, 125.53, 124.46, 121.36, 117.41, 82.49, 67.17, 66.48 ppm; HRMS (ESI) *m*/*z*: [M + Na]^+^ calcd for C_17_H_13_NO_3_S_2_Na 366.0229; found: 366.0242.

*(5R,6R,8aR)-5-Hydroxy-10,11-dimethoxy-5,6-dihydro-6,8a-epoxybenzo[g]benzo[4,5]isothiazolo[2,3-a]azocine 13,13-dioxide* (**11c**). General procedure **D** was followed on 0.100 g (0.26 mmol) scale of photoprecursor **8c**. After the photochemical reaction (irradiation time = 2 h), the crude product was purified by flash chromatography (SiO_2_, 0–40% ethyl acetate in hexanes) which afforded 83 mg (83%) of photoproduct (**11c**) as a brown solid. ^1^H NMR (500 MHz, DMSO-*d*_6_) *δ* 8.08 (d, *J* = 7.6 Hz, 1H), 7.91–7.86 (m, 1H), 7.85–7.81 (m, 2H), 7.73 (dd, *J* = 5.9, 3.4 Hz, 1H), 7.65 (d, *J* = 7.4 Hz, 1H), 7.35 (dd, *J* = 6.1, 3.4 Hz, 2H), 6.79 (dd, *J* = 5.7, 1.8 Hz, 1H), 6.30 (d, *J* = 6.5 Hz, 1H), 6.12 (d, *J* = 5.7 Hz, 1H), 5.00 (s, 1H), 4.97 (dd, *J* = 6.5, 3.3 Hz, 1H) ppm; ^13^C NMR (126 MHz, DMSO-*d*_6_) *δ* 138.95, 137.44, 135.61, 135.13, 134.64, 132.30, 130.41, 129.03, 128.08, 126.95, 126.77, 126.26, 125.51, 121.37, 99.35, 84.95, 74.51 ppm; HRMS (ESI) *m*/*z*: [M + H]^+^ calcd for C_19_H_18_NO_6_S 388.0849; found: 388.0857.

*(4bR,7aS,8R)-8-Hydroxy-2,3-dimethoxy-7a,8-dihydrobenzo[4,5]isothiazolo[2,3-a]thieno[2,3-b]quinoline 14,14-dioxide* (**11d**). General procedure **D** was followed on 0.100 g (0.24 mmol) scale of photoprecursor **8d**. After the photochemical reaction (irradiation time = 8 h), the crude product was purified by flash chromatography (SiO_2_, 0–60% ethyl acetate in hexanes) which afforded 78 mg (78%) of photoproduct (**11d**) as a white amorphous solid. ^1^H NMR (500 MHz, CDCl_3_) *δ* 7.55 (dd, *J* = 6.4, 2.7 Hz, 1H), 7.50 (dd, *J* = 5.8, 3.0 Hz, 1H), 7.41 (dd, *J* = 6.2, 2.8 Hz, 2H), 7.24 (s, 1H), 7.13 (s, 1H), 6.23 (dd, *J* = 6.7, 2.8 Hz, 1H), 5.39 (dd, *J* = 6.6, 2.2 Hz, 1H), 5.04 (d, *J* = 5.7 Hz, 1H), 4.39 (dt, *J* = 5.5, 2.6 Hz, 1H), 4.03 (s, 3H), 4.00 (s, 3H) ppm; ^13^C NMR (126 MHz, CDCl_3_) *δ* 154.15, 151.26, 136.48, 132.70, 131.22, 128.24, 128.09, 127.28, 125.94, 125.88, 124.39, 117.56, 106.66, 102.21, 82.58, 67.11, 66.24, 56.66, 56.59 ppm; HRMS (ESI) *m*/*z*: [M + Na]^+^ calcd for C_19_H_17_NO_5_S_2_Na 426.0440; found: 426.0442.

*(4bR,7aS,8S)-8-Hydroxy-8-methyl-7a,8-dihydrobenzo[4,5]isothiazolo[2,3-a]furo[2,3-b]quinoline 14,14-dioxide* (**11e**). General procedure **D** was followed on 0.100 g (0.29 mmol) scale of photoprecursor **10a**. After the photochemical reaction (irradiation time = 5 h), the crude product was purified by flash chromatography (SiO_2_, 0–40% ethyl acetate in hexanes) which afforded 79 mg (79%) of photoproduct (**11e**) and 14 mg (14%) of photoproduct (**11ea**) as a white solid. ^1^H NMR (500 MHz, CDCl_3_) *δ* 7.90 (d, *J* = 7.9 Hz, 1H), 7.81 (t, *J* = 7.8 Hz, 1H), 7.69 (q, *J* = 8.3, 7.8 Hz, 3H), 7.49 (q, *J* = 7.8 Hz, 2H), 7.38 (t, *J* = 7.5 Hz, 1H), 6.47–6.42 (m, 1H), 4.83 (s, 1H), 4.20 (s, 1H), 2.99 (s, 1H; OH), 1.75 (s, 3H) ppm; ^13^C NMR (126 MHz, CDCl_3_) *δ* 147.12, 138.79, 135.71, 134.40, 133.14, 131.81, 131.15, 129.94, 127.79, 125.90, 125.71, 124.51, 121.55, 100.29, 97.35 70.39, 63.44, 24.59 ppm; HRMS (ESI) *m*/*z*: [M + Na]^+^ calcd for C_18_H_15_NO_4_SNa 364.0614; found: 364.0599.

*(5R,6S,8aS)-5-Hydroxy-5-methyl-5,6-dihydro-6,8a-epoxybenzo[g]benzo[4,5]isothiazolo[2,3-a]azocine 13,13-dioxide* (**11ea**). ^1^H NMR (500 MHz, CDCl_3_) *δ* 7.96 (d, *J* = 7.3 Hz, 1H), 7.75 (dt, *J* = 13.0, 7.8 Hz, 3H), 7.64 (d, *J* = 7.8 Hz, 1H), 7.59 (d, *J* = 7.2 Hz, 1H), 7.37 (dt, *J* = 18.7, 7.4 Hz, 2H), 6.62 (d, *J* = 5.5 Hz, 1H), 5.88 (d, *J* = 5.8 Hz, 1H), 4.91 (s, 1H), 3.62 (s, 1H; OH), 1.81 (s, 3H) ppm; ^13^C NMR (126 MHz, CDCl_3_) *δ* 138.62, 138.33, 135.98, 135.24, 133.54, 131.35, 130.13, 129.23, 129.02, 128.20, 127.74, 125.47, 124.35, 121.36, 99.40, 89.60, 78.06, 24.62 ppm; HRMS (ESI) *m*/*z*: [M + Na]^+^ calcd for C_18_H_15_NO_4_SNa 364.0614; found: 364.0593.

*(4bR,7aS,8R)-8-Hydroxy-8-methyl-7a,8-dihydrobenzo[4,5]isothiazolo[2,3-a]thieno[2,3-b]quinoline 14,14-dioxide* (**11f**). General procedure **D** was followed on 0.100 g (0.28 mmol) scale of photoprecursor **10b**. After the photochemical reaction (irradiation time = 3 h), the crude product was purified by flash chromatography (SiO_2_, 0–60% ethyl acetate in hexanes) which afforded 78 mg (78%) of photoproduct (**11f**) as a white solid. ^1^H NMR (500 MHz, CDCl_3_) *δ* 7.82–7.74 (m, 3H), 7.61 (dd, *J* = 13.3, 7.4 Hz, 2H), 7.47 (d, *J* = 7.5 Hz, 2H), 7.36 (t, *J* = 7.7 Hz, 1H), 6.22 (dd, *J* = 6.9, 3.1 Hz, 1H), 5.13 (d, *J* = 6.7 Hz, 1H), 4.36 (s, 1H), 1.80 (s, 3H) ppm; ^13^C NMR (126 MHz, CDCl_3_) *δ* 142.08, 136.61, 134.03, 132.53, 132.21, 130.03, 129.97, 127.84, 127.16, 126.31, 125.75, 125.62, 121.48, 118.29, 82.62, 71.21, 71.02, 25.61 ppm; HRMS (ESI) *m*/*z*: [M + Na]^+^ calcd for C_18_H_15_NO_3_S_2_Na 380.0385; found: 380.0392.

*(3aR,15aS,15bS)-15a-Hydroxy-14,15,15a,15b-tetrahydro-13H-benzo[de]benzo[4,5]isothiazolo[2,3-a]furo[2,3-b]quinoline 8,8-dioxide* (**11g**). General procedure **D** was followed on 0.100 g (0.27 mmol) scale of photoprecursor **10c**. After the photochemical reaction (irradiation time = 2 h), the crude product was purified by flash chromatography (SiO_2_, 0–50% ethyl acetate in hexanes) which afforded 91 mg (91%) of photoproduct (**11g**) as a pale-yellow solid. ^1^H NMR (500 MHz, CDCl_3_) δ 7.89 (d, *J* = 7.8 Hz, 1H), 7.81 (t, *J* = 7.6 Hz, 1H), 7.75–7.66 (m, 2H), 7.50 (d, *J* = 7.7 Hz, 1H), 7.35 (t, *J* = 7.8 Hz, 1H), 7.16 (d, *J* = 7.8 Hz, 1H), 6.47 (t, *J* = 3.0 Hz, 1H), 4.87 (t, *J* = 2.7 Hz, 1H), 4.20 (t, *J* = 2.6 Hz, 1H), 3.03 (s, 1H; OH), 2.87–2.81 (m, 1H), 2.75 (ddd, *J* = 16.9, 12.1, 5.2 Hz, 1H), 2.10–2.00 (m, 2H), 1.91–1.85 (m, 1H), 1.84–1.76 (m, 1H) ppm; ^13^C NMR (126 MHz, CDCl_3_) δ 146.94, 139.24, 138.93, 134.35, 133.08, 131.69, 131.07, 130.77, 129.11, 128.63, 124.54, 123.22, 121.55, 100.52, 97.43, 68.61, 61.98, 34.59, 29.54, 18.15 ppm; HRMS (ESI) *m*/*z*: [M + Na]^+^ calcd for C_20_H_17_NO_4_SNa 390.0770; found: 390.0780.

#### 4.2.5. Postphotochemical Modifications (General Procedure E)

Typically, 1 equiv. photoproduct and 1 equiv. 1,3-dicarbonyl compound were dissolved in 0.7 mL dry acetonitrile. To this solution, 0.08 equiv. L-proline and 1.3 equiv. 37% aqueous formaldehyde solution were added. The reaction was stirred at ambient temperature until complete consumption of the photoproduct, as determined by ^1^H NMR analysis. The reaction was diluted with water and extracted with EtOAc. The organic layer was separated, dried over Na_2_SO_4_, and concentrated under vacuum. The mixture was then purified by flash chromatography.

*(5aR,17aS,17bS)-17a-Hydroxy-2-methylene-1,2,4a,16,17,17a,17b,17c-octahydro-3H,15H-benzo[de]benzo[4,5]isothiazolo[2,3-a]pyrano[3′,2′:4,5]furo[2,3-b]quinolin-3-one 10,10-dioxide* (**12**). General procedure **E** was followed using 50 mg (**11g**; 0.13 mmol), 20 mg of Meldrum’s acid (0.13 mmol), 3.0 mg L-proline (0.04 mmol), and 0.03 mL formaldehyde solution (37% *w*/*w*) in water (0.11 mmol) to generate the title compound **12** (33 mg, 54%). ^1^H NMR (500 MHz, CDCl_3_) δ 7.84 (d, *J* = 7.9 Hz, 1H), 7.79 (t, *J* = 7.7 Hz, 1H), 7.66 (t, *J* = 7.6 Hz, 1H), 7.61 (d, *J* = 7.9 Hz, 1H), 7.48 (d, *J* = 7.8 Hz, 1H), 7.42 (t, *J* = 7.8 Hz, 1H), 7.21 (d, *J* = 7.7 Hz, 1H), 6.54 (s, 1H), 5.84 (d, *J* = 5.7 Hz, 1H), 5.77 (s, 1H), 3.32 (d, *J* = 10.5 Hz, 1H), 3.02 (s, 1H; OH), 2.95–2.85 (m, 2H), 2.85–2.74 (m, 2H), 2.40 (dtd, *J* = 11.1, 5.7, 3.0 Hz, 1H), 2.04–1.93 (m, 2H), 1.92–1.84 (m, 1H), 1.72 (td, *J* = 13.1, 2.8 Hz, 1H) ppm; ^13^C NMR (126 MHz, CDCl_3_) δ 164.41, 139.52, 138.85, 134.83, 132.54, 131.58, 131.38, 131.04, 130.04, 129.90, 129.22, 129.06, 124.83, 124.01, 121.49, 103.54, 98.87, 69.47, 58.18, 40.54, 34.87, 31.24, 29.51, 17.86 ppm.

*(5aR,17aS,17bS)-17a-Hydroxy-2-methylene-1,2,4a,16,17,17a,17b,17c-octahydro-3H,15H-benzo[de]benzo[4,5]isothiazolo[2,3-a]pyrano[3′,2′:4,5]furo[2,3-b]quinolin-3-one 10,10-dioxide* (**13**). General procedure **E** was followed using 50 mg (**11g**; 0.13 mmol), 22 mg of 1,3-dimethylbarbituric acid (0.13 mmol), 3.0 mg L-proline (0.04 mmol), and 0.03 mL formaldehyde solution (37% *w*/*w*) in water (0.11 mmol) to generate the title compound **13** (32 mg, 43%). ^1^H NMR (500 MHz, CDCl_3_) δ 7.88 (d, *J* = 7.7 Hz, 1H), 7.76 (t, *J* = 7.6 Hz, 1H), 7.69 (t, *J* = 7.6 Hz, 1H), 7.50 (d, *J* = 7.8 Hz, 1H), 7.45–7.38 (m, 2H), 7.21 (d, *J* = 7.8 Hz, 1H), 5.77 (d, *J* = 4.8 Hz, 1H), 3.46 (s, 3H), 3.42 (s, 3H), 3.27 (d, *J* = 11.2 Hz, 1H), 3.02 (d, *J* = 17.1 Hz, 1H), 2.88 (d, *J* = 16.5 Hz, 1H), 2.77–2.69 (m, 2H), 2.47–2.39 (m, 1H), 1.99 (d, *J* = 11.0 Hz, 1H), 1.92 (s, 3H) ppm; ^13^C NMR (126 MHz, CDCl_3_) δ 162.59, 153.72, 151.03, 139.83, 138.91, 134.19, 133.10, 131.13, 131.00, 129.82, 129.51, 128.84, 124.10, 123.05, 121.77, 103.55, 99.41, 83.96, 69.70, 57.53, 39.90, 35.40, 29.68, 28.80, 28.17, 19.99, 18.03 ppm.

## 5. Conclusions

Aromatic sulfonamides, readily accessible via a modular “assembly” from common building blocks/diversity inputs, undergo excited state intramolecular proton transfer (ESIPT) yielding N-sulfonyl aza-*o*-xylylenes, which are capable of intramolecular [4+2] and [4+4] cycloadditions with tethered unsaturated pendants, most likely in the triplet manifold. The primary photoproducts are amenable to experimentally simple postphotochemical ground state reactions, offering opportunities to introduce additional diversity inputs and further grow molecular complexity as quantified by the Böttcher complexity indices. These findings expand the scope of ESIPT-based synthetic approaches and, generally, enhance the toolchest of photoassisted synthetic chemistry.

## Figures and Tables

**Table 1:** Photoprecursors, primary photoproducts (isolated yields).

Entry	Photoprecursor	Photoproduct(s)	Isolated Yields
**1**	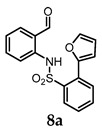	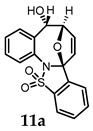	**87%**
**2**	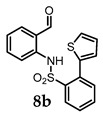	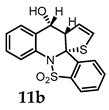	**81%**
**3**	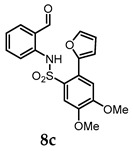	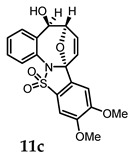	**83%**
**4**	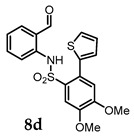	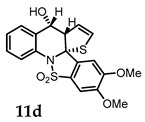	**78%**
**5**	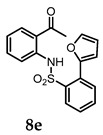	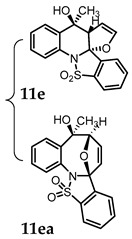	**79%**
			**14%**
**6**	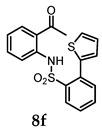	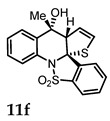	**78%**
**7**	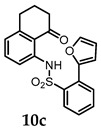	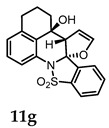	**91%**

**Table 2:** UV absorption of photoprecursors at 365nm.

Entry	Photoprecursor	Absorbance (A)	Concentration (M)	Extinction Coefficient (M^−1^ cm^−1^)
1	**8a**	0.051	6.11 × 10^−5^	0.83 × 10^3^
2	**8b**	0.084	5.82 × 10^−5^	1.44 × 10^3^
3	**8c**	0.052	5.16 × 10^−5^	1.00 × 10^3^
4	**8d**	0.088	4.95 × 10^−5^	1.77 × 10^3^
5	**8e**	0.066	5.86 × 10^−5^	1.12 × 10^3^
6	**8f**	0.080	5.60 × 10^−5^	1.42 × 10^3^
7	**10c**	0.077	5.44 × 10^−5^	1.41 × 10^3^

**Table 3:** Typical DU8ML computations exemplified by primary photoproduct **11g** (rmsd = 0.99 ppm).

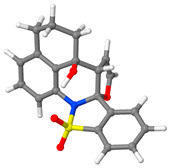			
Atom Type	Exp δ_13C_	Calcd δ_13C_	∆δ_13C_
C	139.24	139.54	0.30
C	138.93	139.61	0.68
C	133.08	134.83	1.75
C	131.69	131.33	−0.36
C	130.77	130.05	−0.72
C	97.43	98.48	1.05
C	68.61	70.73	2.12
CH	146.94	146.34	−0.60
CH	134.35	135.92	1.57
CH	131.07	130.51	−0.56
CH	129.11	129.38	0.27
CH	128.63	128.61	−0.02
CH	124.54	125.49	0.95
CH	123.22	124.98	1.76
CH	121.55	121.99	0.44
CH	100.52	99.66	−0.86
CH	61.98	63.12	1.14
CH2	34.59	34.53	−0.06
CH2	29.54	29.33	−0.21
CH2	18.15	18.77	0.62

## Data Availability

The data presented in this study are available in the Supplementary Materials.

## Supplementary Materials

The following supporting information can be downloaded at: https://www.mdpi.com/article/10.3390/molecules28186549/s1, NMR spectra.
