# Supplementary material for: Complexity-Building ESIPT-Assisted Synthesis of Fused Polyheterocyclic Sulfonamides
Source: Molecules. 2023 Sep 10;28(18):6549. doi: 10.3390/molecules28186549 (PMC10534920; doi:10.3390/molecules28186549)
Supplement: Supplementary file 1 [file molecules-28-06549-s001.zip › molecules-2509475-supplementary.pdf]

Supporting Information for

Complexity-Building ESIPT-Assisted Synthesis of Fused Polyheterocyclic Sulfonamides.

Srinivas Beduru and Andrei G. Kutateladze*

Department of Chemistry and Biochemistry, University of Denver, USA, 80208

*Correspondence: Andrei.Kutateladze@du.edu

**NMR spectra**	**Page**

*N-(2-Formylphenyl)-2-(furan-2-yl)benzenesulfonamide (**8a**)*

^{1}H NMR spectrum S5

^{13}C NMR spectrum S6

*N-(2-Formylphenyl)-2-(thiophen-2-yl)benzenesulfonamide (**8b**)*

^{1}H NMR spectrum S7

^{13}C NMR spectrum S8

*N-(2-Formylphenyl)-2-(furan-2-yl)-4,5-dimethoxybenzenesulfonamide (**8c**)*

^{1}H NMR spectrum S9

^{13}C NMR spectrum S10

*N-(2-Formylphenyl)-4,5-dimethoxy-2-(thiophen-2-yl)benzenesulfonamide (**8d**)*

^{1}H NMR spectrum S11

^{13}C NMR spectrum S12

*2-(Furan-2-yl)-N-(8-oxo-5,6,7,8-tetrahydronaphthalen-1-yl)benzenesulfonamide (**10c**)*

^{1}H NMR spectrum S13

^{13}C NMR spectrum S14

*(5S,6S,8aS)-5-Hydroxy-5,6-dihydro-6,8a-epoxybenzo[g]benzo[4,5]isothiazolo[2,3-a]azocine 13,13-dioxide (**11a**)*

^{1}H NMR spectrum S15

^{13}C NMR spectrum S16

HSQC spectrum S17

*(4bR,7aS,8R)-8-Hydroxy-7a,8-dihydrobenzo[4,5]isothiazolo[2,3-a]thieno[2,3-b]quinoline 14,14-dioxide (**11b**)*

^{1}H NMR spectrum S18

^{13}C NMR spectrum S19

HSQC spectrum S20

*(5R,6R,8aR)-5-Hydroxy-10,11-dimethoxy-5,6-dihydro-6,8a-epoxybenzo[g]benzo[4,5]isothiazolo[2,3-a]azocine 13,13-dioxide (**11c**)*

^{1}H NMR spectrum S21

^{13}C NMR spectrum S22
HSQC spectrum S23
*(4bR,7aS,8R)-8-Hydroxy-2,3-dimethoxy-7a,8-dihydrobenzo[4,5]isothiazolo[2,3-a]thieno[2,3-b]quinoline 14,14-dioxide (**11d**)*
^{1}H NMR spectrum S24
^{13}C NMR spectrum S25
HSQC spectrum S26
*(4bR,7aS,8S)-8-Hydroxy-8-methyl-7a,8-dihydrobenzo[4,5]isothiazolo[2,3-a]furo[2,3-b]quinoline 14,14-dioxide (**11e**)*
^{1}H NMR spectrum S27
^{13}C NMR spectrum S28
HSQC spectrum S29
*(5R,6S,8aS)-5-Hydroxy-5-methyl-5,6-dihydro-6,8a-epoxybenzo[g]benzo[4,5]isothiazolo[2,3-a]azocine 13,13-dioxide (**11ea**)*
^{1}H NMR spectrum S30
^{13}C NMR spectrum S31
HSQC spectrum S32
*(4bR,7aS,8R)-8-Hydroxy-8-methyl-7a,8-dihydrobenzo[4,5]isothiazolo[2,3-a]thieno[2,3-b]quinoline 14,14-dioxide (**11f**)*
^{1}H NMR spectrum S33
^{13}C NMR spectrum S34
HSQC spectrum S35
*(3aR,15aS,15bS)-15a-Hydroxy-14,15,15a,15b-tetrahydro-13H-benzo[de]benzo[4,5]isothiazolo[2,3-a]furo[2,3-b]quinoline 8,8-dioxide (**11g**)*
^{1}H NMR spectrum S36
^{13}C NMR spectrum S37
HSQC spectrum S38
*(5aR,17aS,17bS)-17a-Hydroxy-2-methylene-1,2,4a,16,17,17a,17b,17c-octahydro-3H,15H-benzo[de]benzo[4,5]isothiazolo[2,3-a]pyrano[3',2':4,5]furo[2,3-b]quinolin-3-one 10,10-dioxide (**12**)*
^{1}H NMR spectrum S39
^{13}C NMR spectrum S40
HSQC spectrum S41

*(5aR,17aS,17bS)-17a-Hydroxy-2-methylene-1,2,4a,16,17,17a,17b,17c-octahydro-3H,15H-benzo[de]benzo[4,5]isothiazolo[2,3-a]pyrano[3',2':4,5]furo[2,3-b]quinolin-3-one 10,10-dioxide (**13**)*

^{1}H NMR spectrum S42

^{13}C NMR spectrum S43

HSQC spectrum S44

NOESY spectrum S45

UV Spectra of photoprecursors S46

^{1}H NMR spectrum of **8a** (500 MHz, $CDCl_3$)

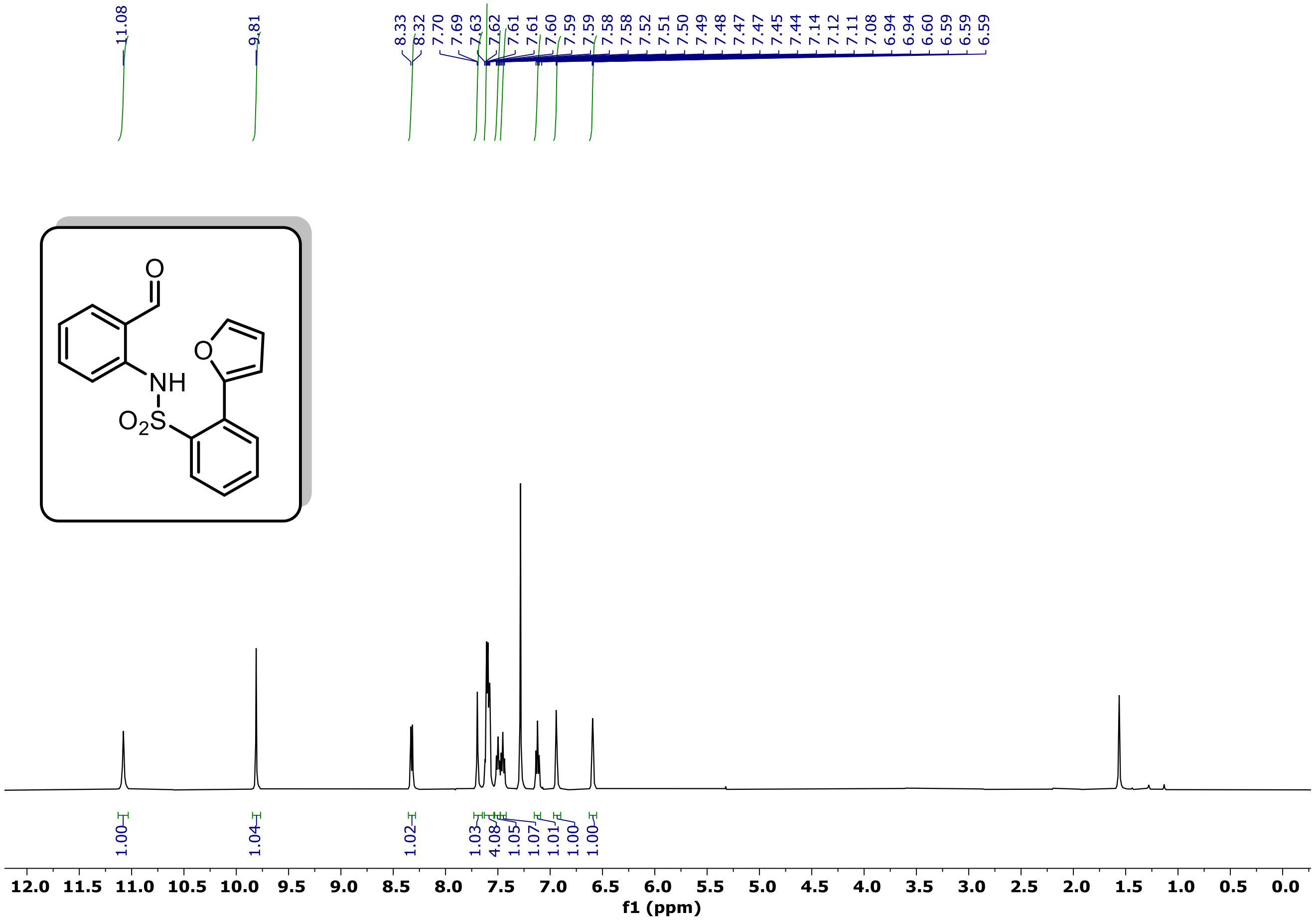

^{13}C NMR spectrum of **8a** (126 MHz, $CDCl_3$)

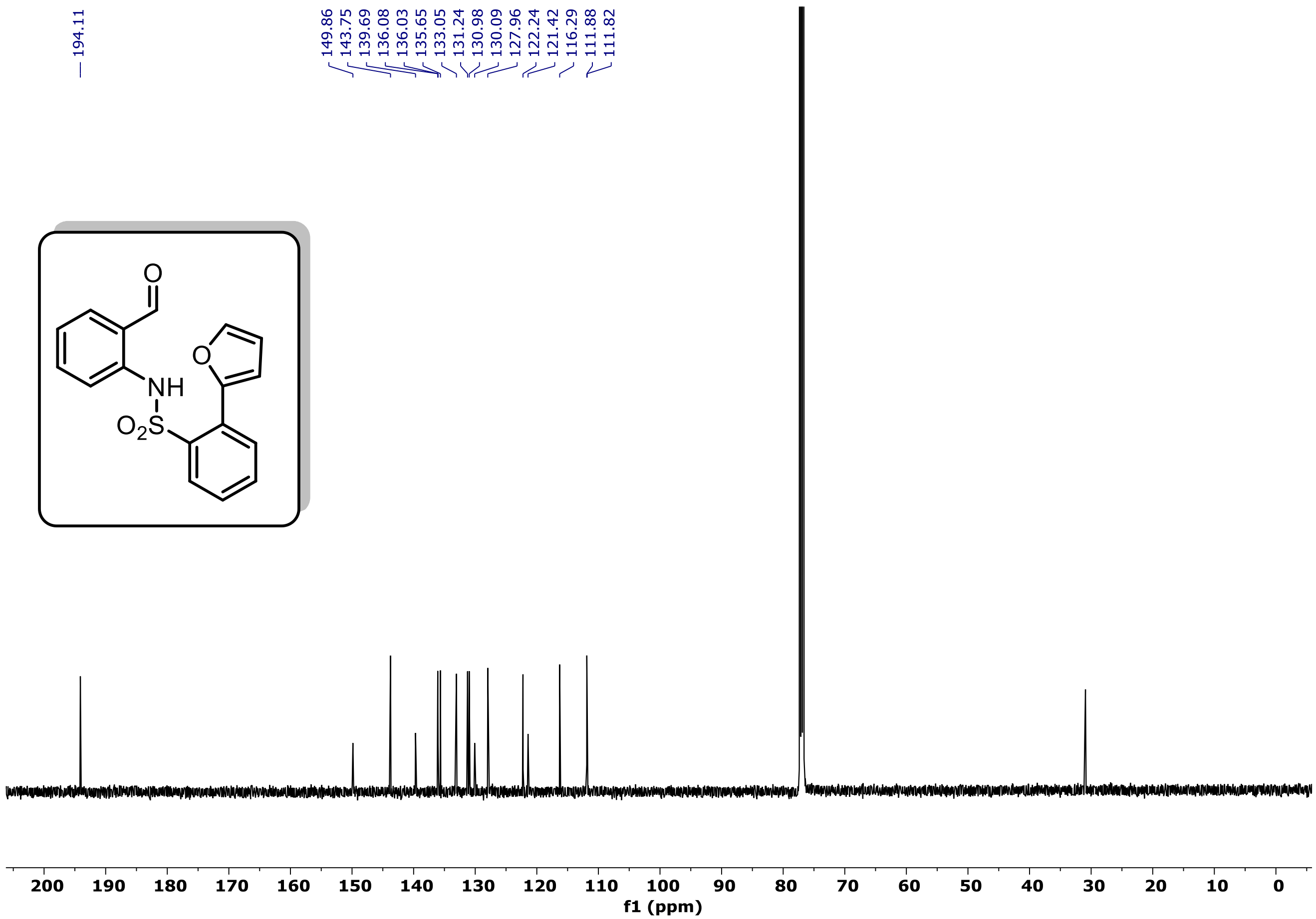

^{1}H NMR spectrum of **8b** (500 MHz, $CDCl_3$)

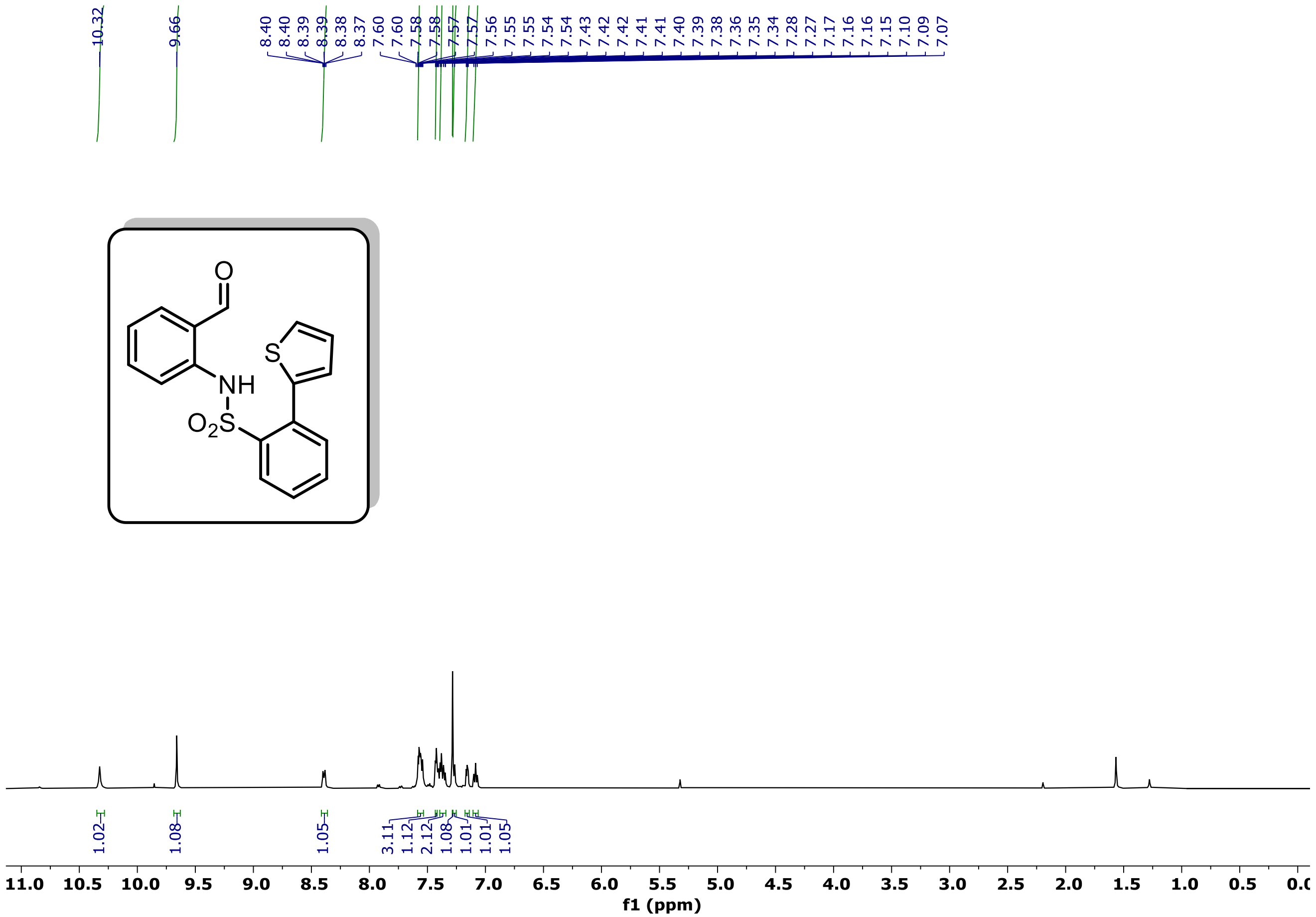

^{13}C NMR spectrum of **8b** (126 MHz, $CDCl_3$)

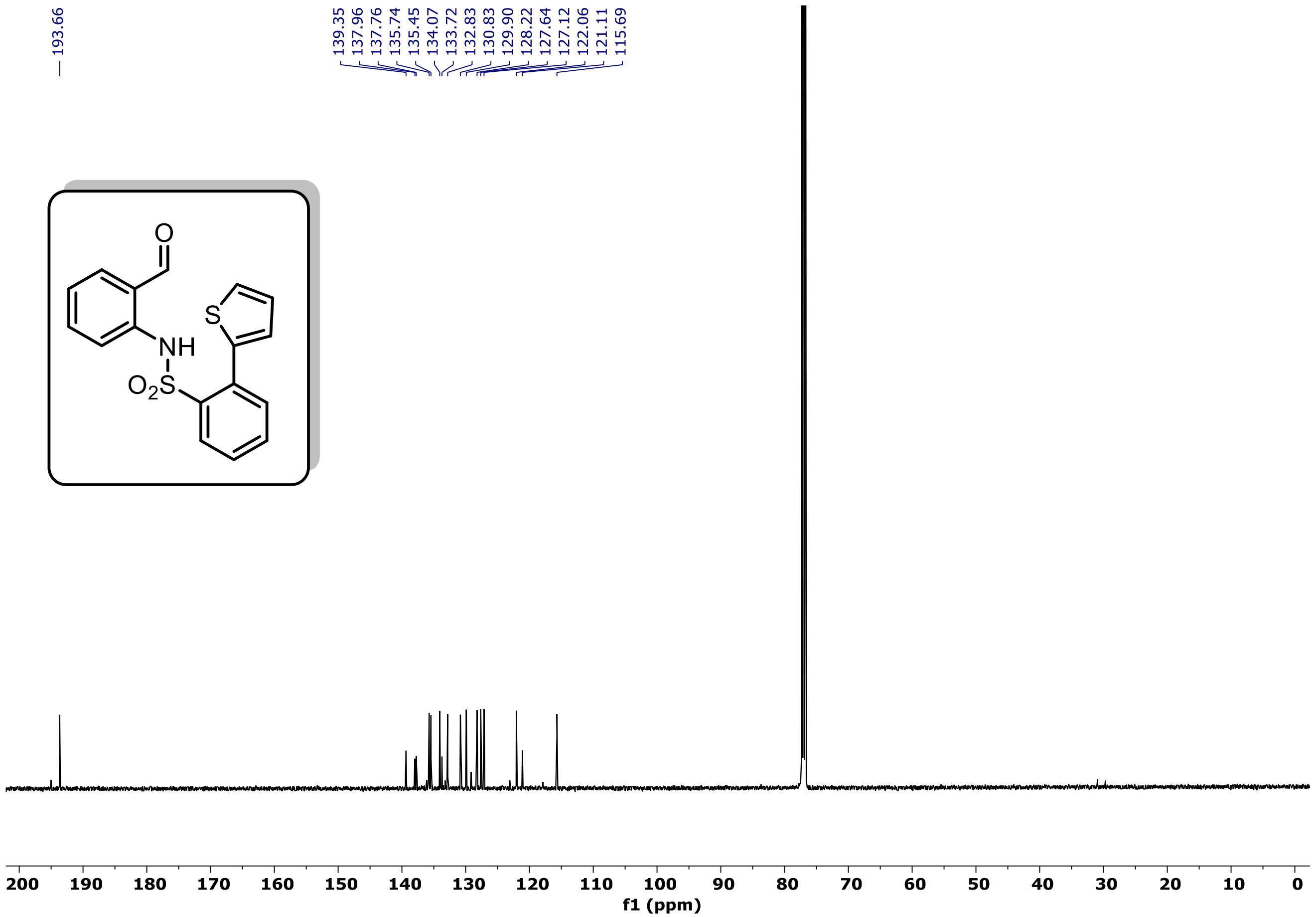

^{1}H NMR spectrum of **8c** (500 MHz, $CDCl_3$)

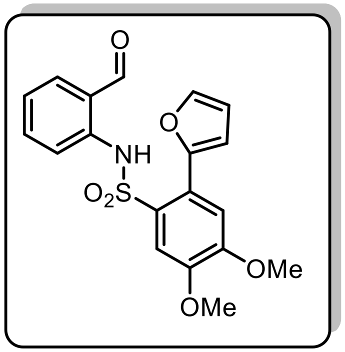

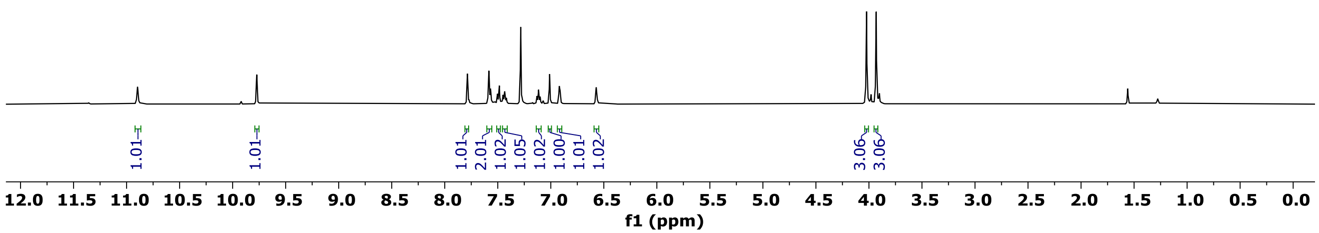

^{13}C NMR spectrum of **8c** (126 MHz, $CDCl_3$)

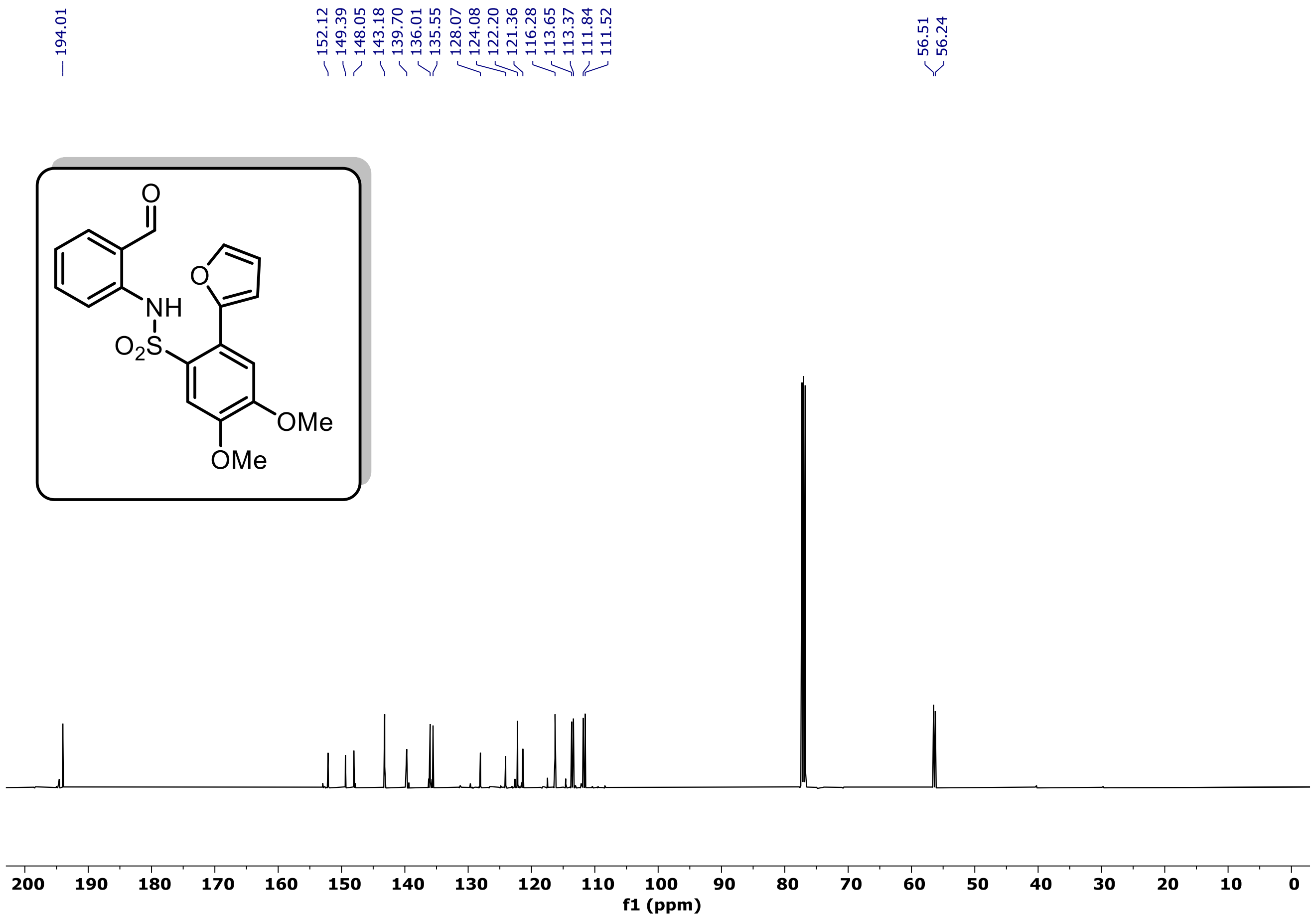

^{1}H NMR spectrum of **8d** (500 MHz, $CDCl_3$)

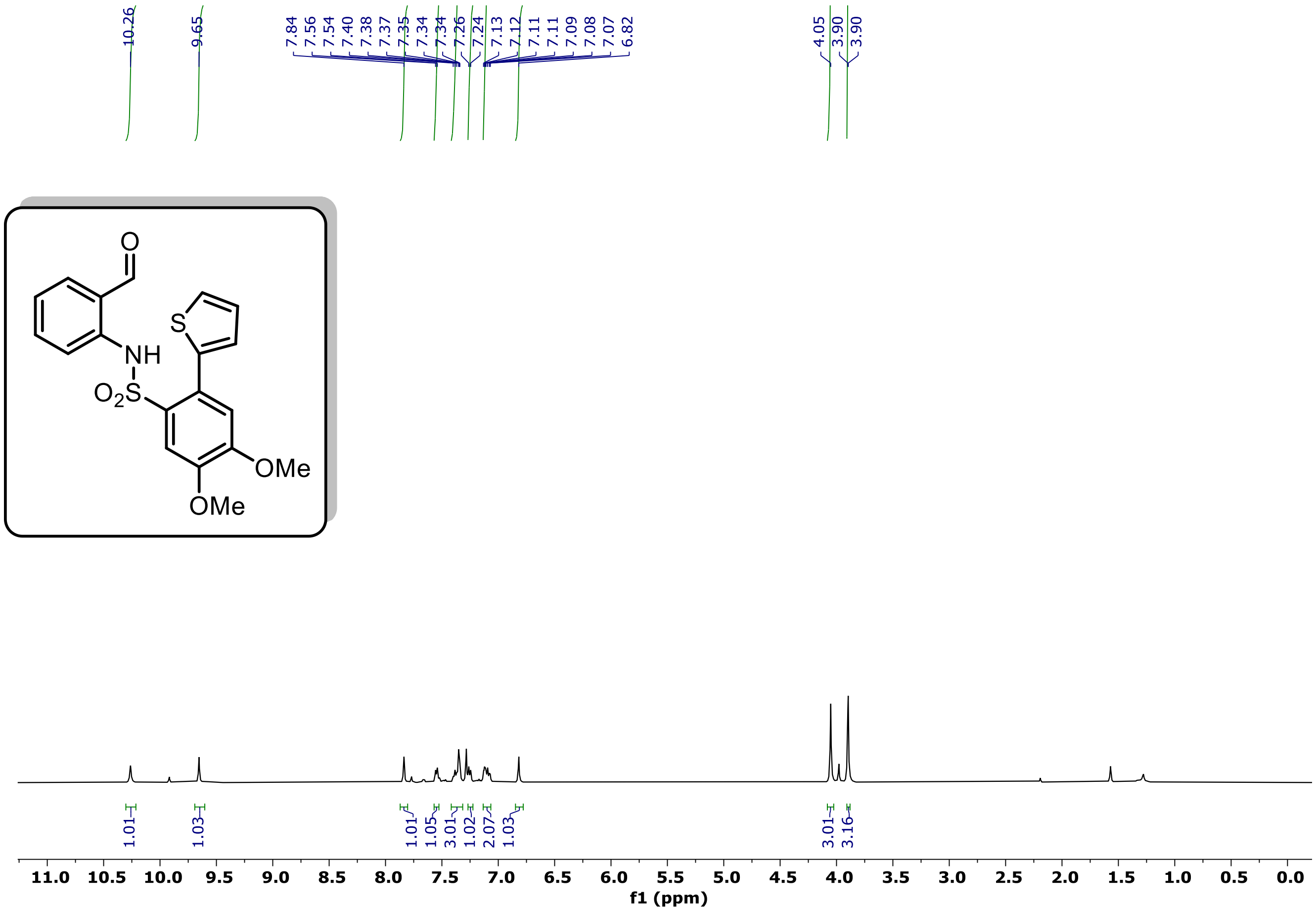

^{13}C NMR spectrum of **8d** (126 MHz, $CDCl_3$)

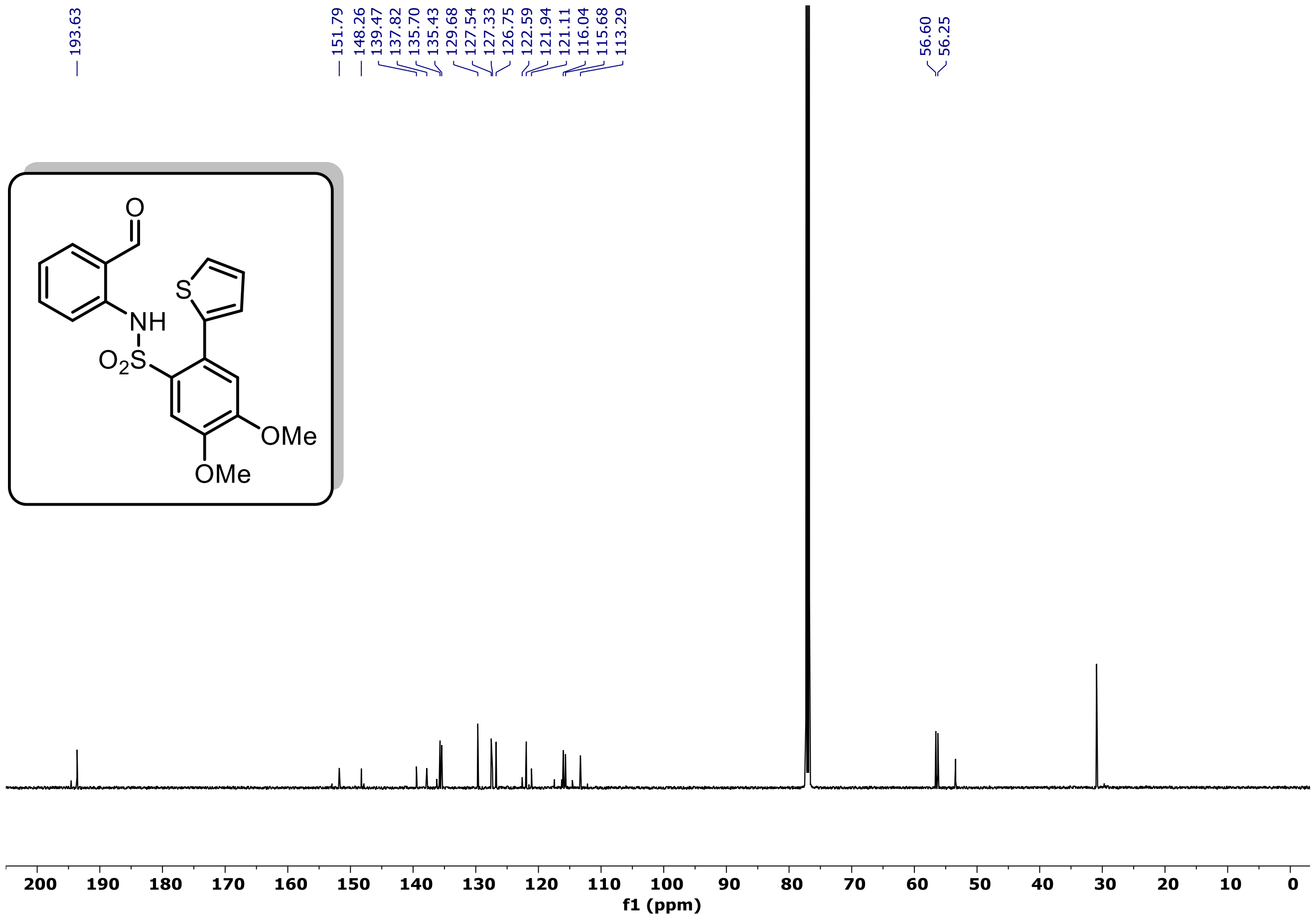

^1H NMR spectrum of **10c** (500 MHz, $CDCl_3$)

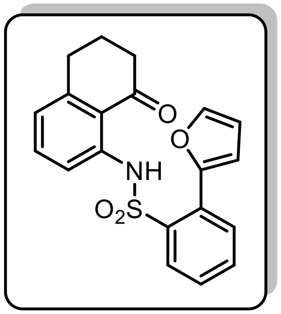

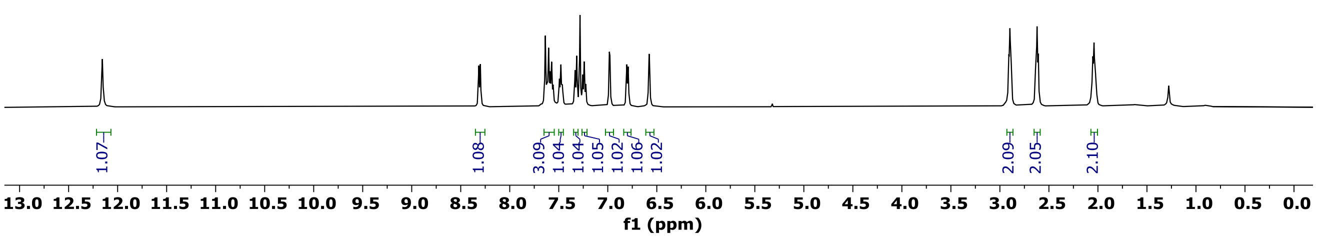

^{13}C NMR spectrum of **10c** (126 MHz, $CDCl_3$)

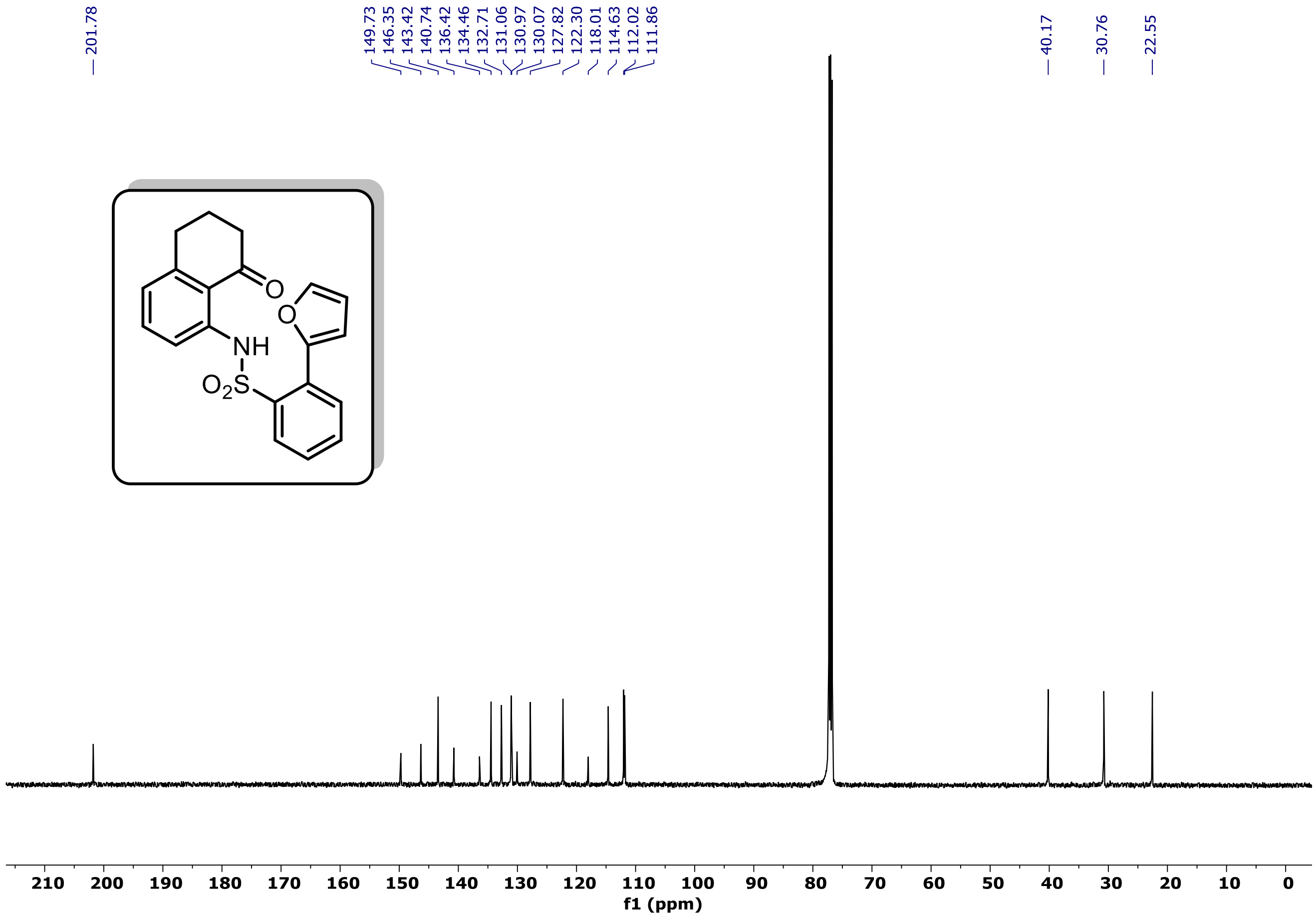

^{1}H NMR spectrum of **11a** (500 MHz, DMSO_d_6)

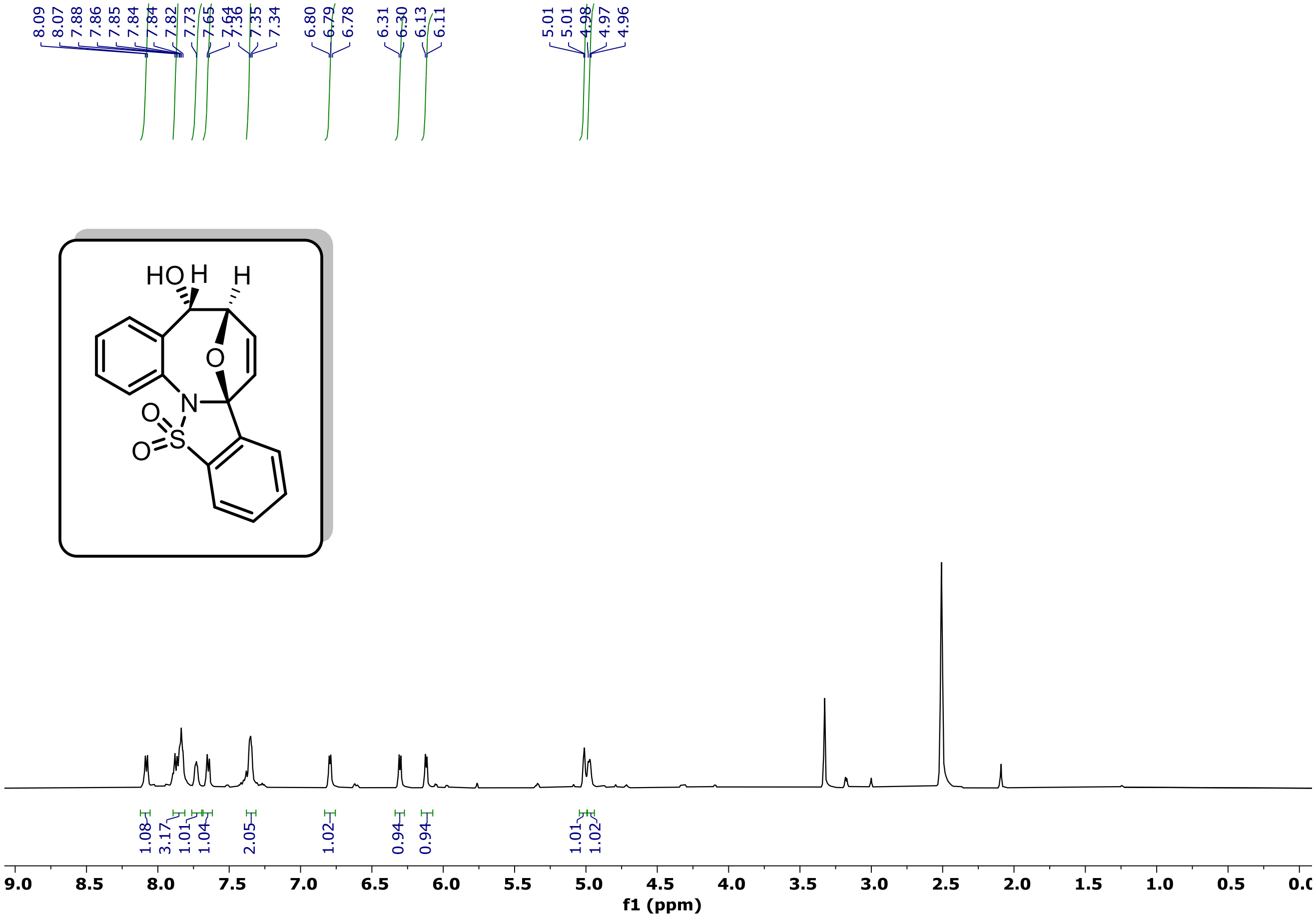

^{13}C NMR spectrum of **11a** (126 MHz, DMSO_d_6)

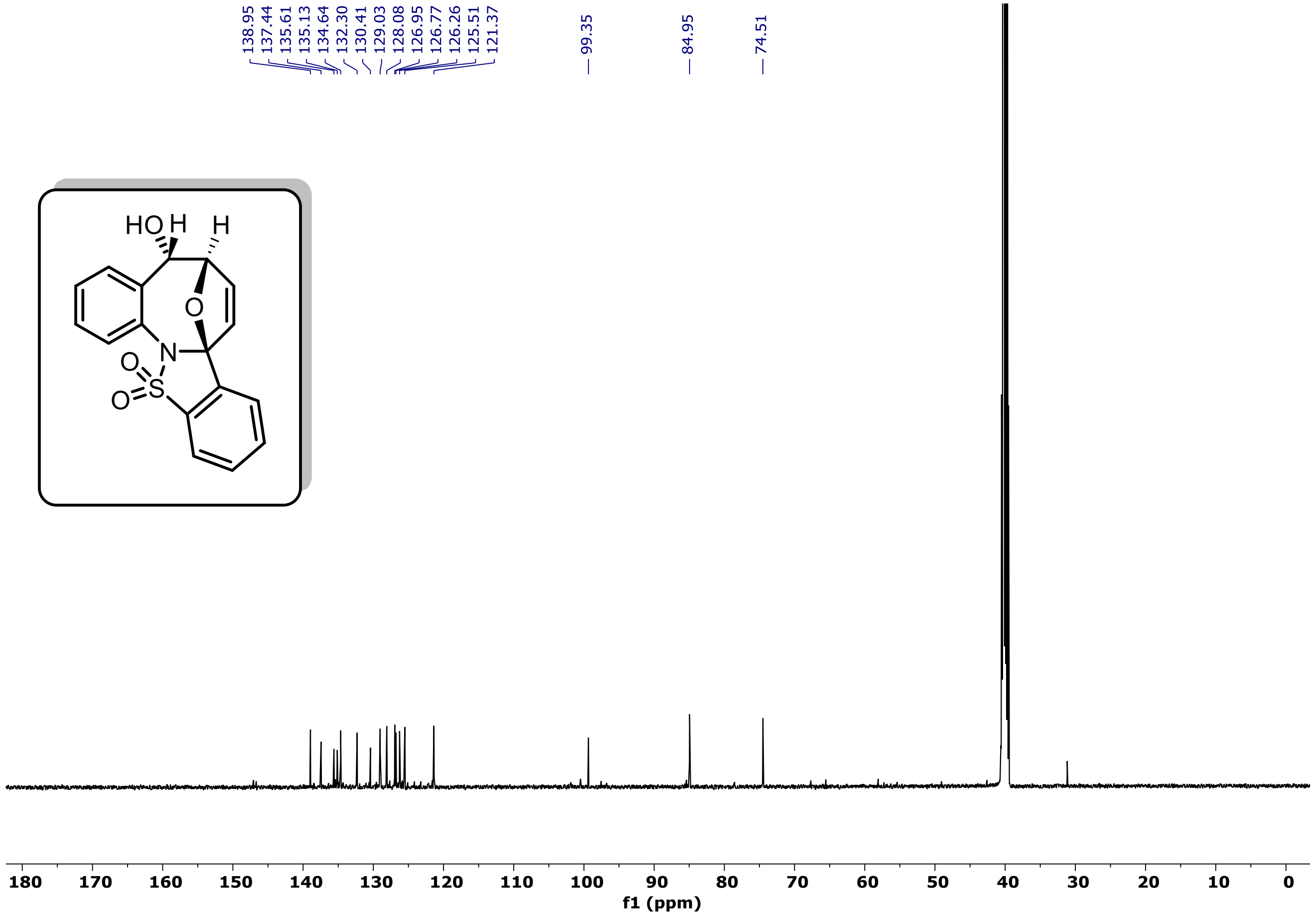

HSQC spectrum of **11a** (DMSO_d_6)

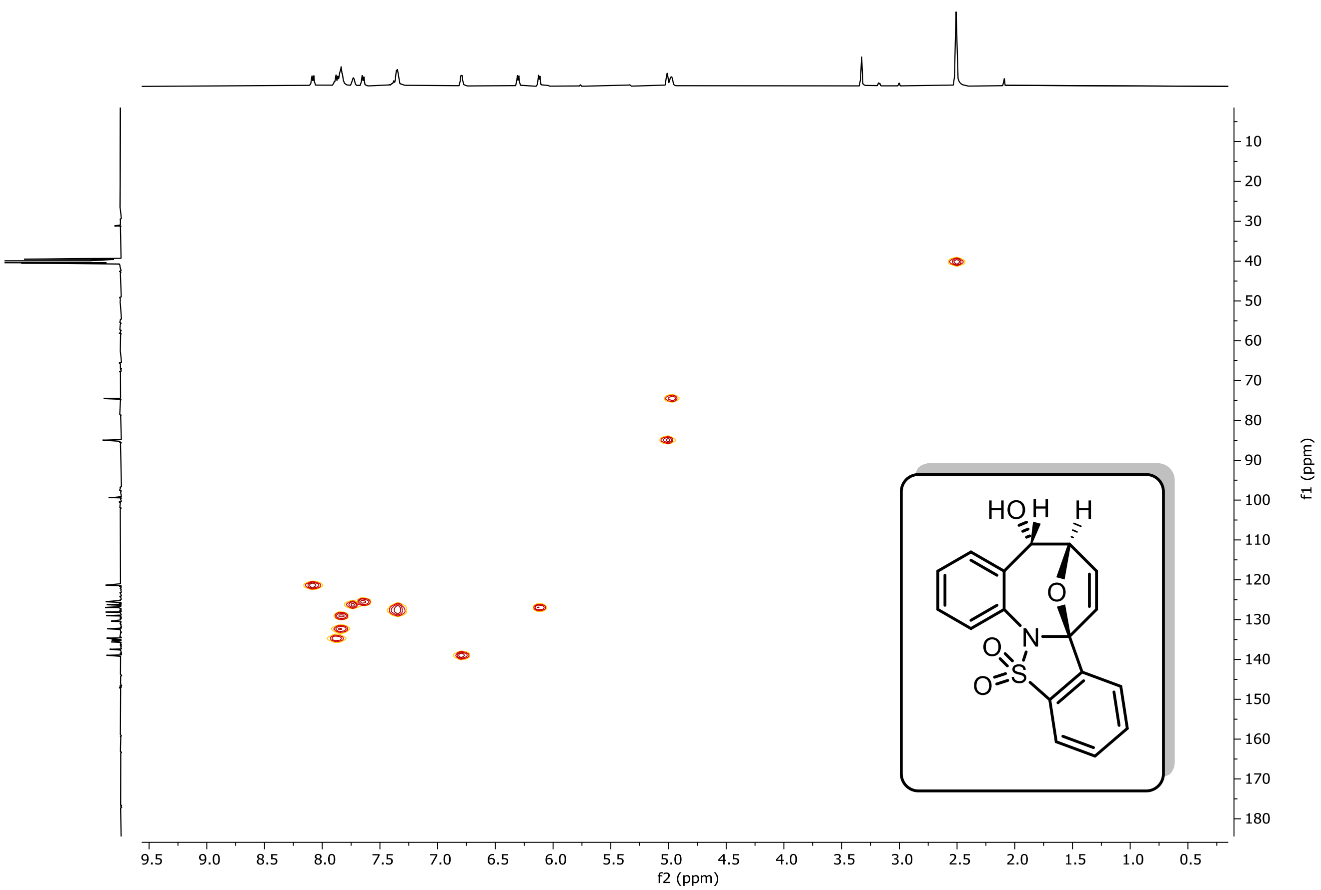

^{1}H NMR spectrum of **11b** (500 MHz, $CDCl_3$)

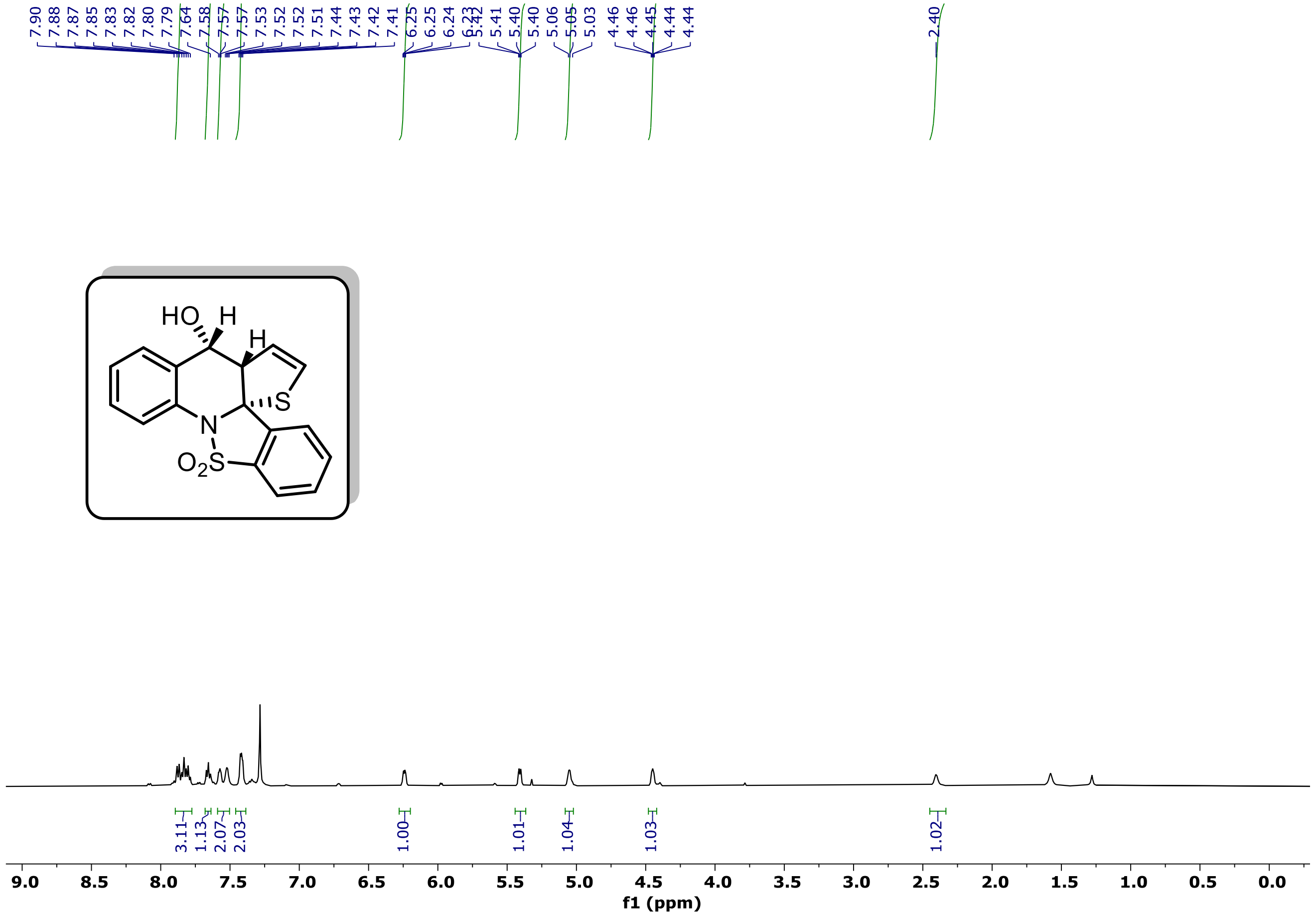

^{13}C NMR spectrum of **11b** (126 MHz, $CDCl_3$)

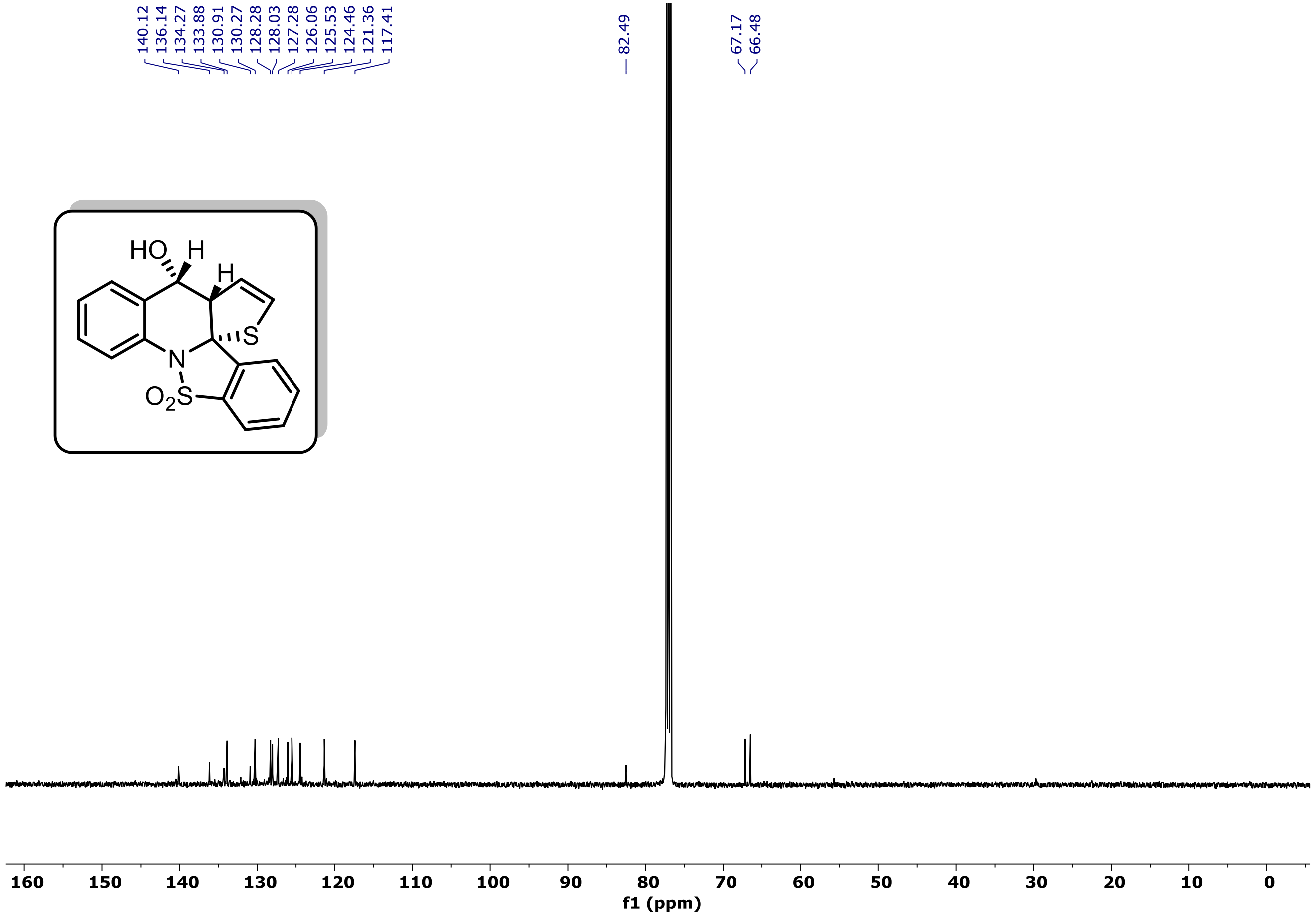

HSQC spectrum of **11b** ($CDCl_3$)

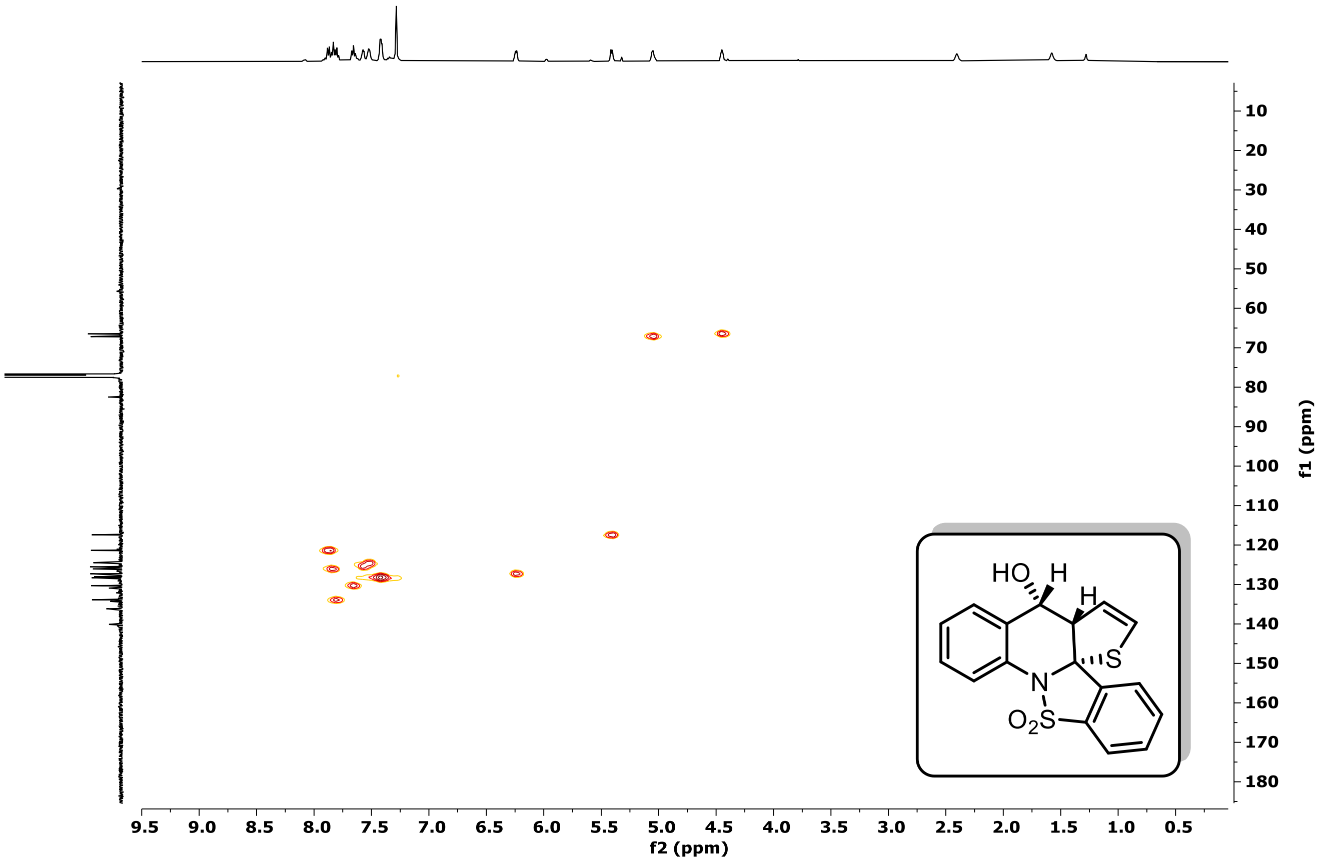

^{1}H NMR spectrum of **11c** (500 MHz, DMSO_d_6)

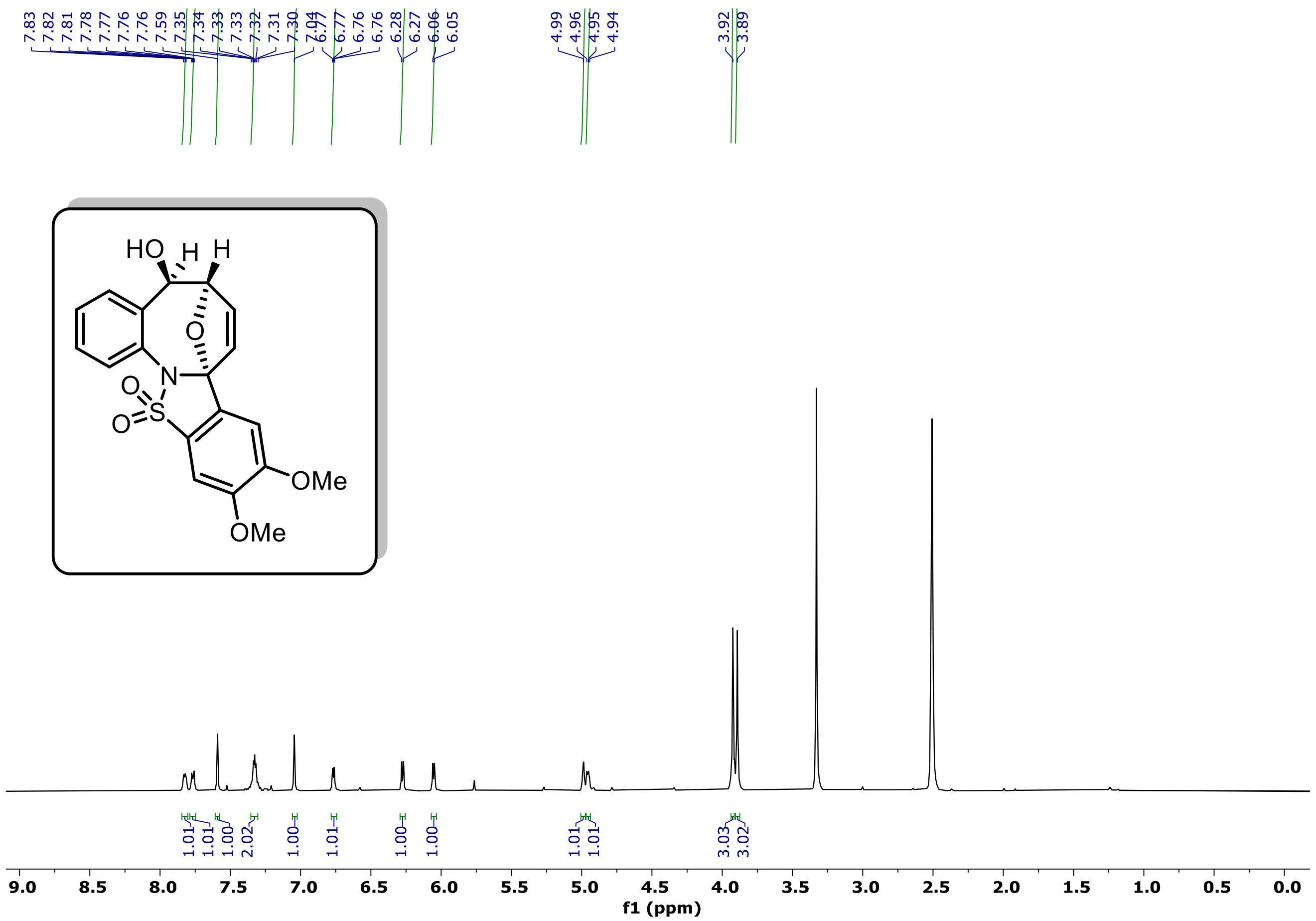

^{13}C NMR spectrum of **11c** (126 MHz, DMSO_d_6)

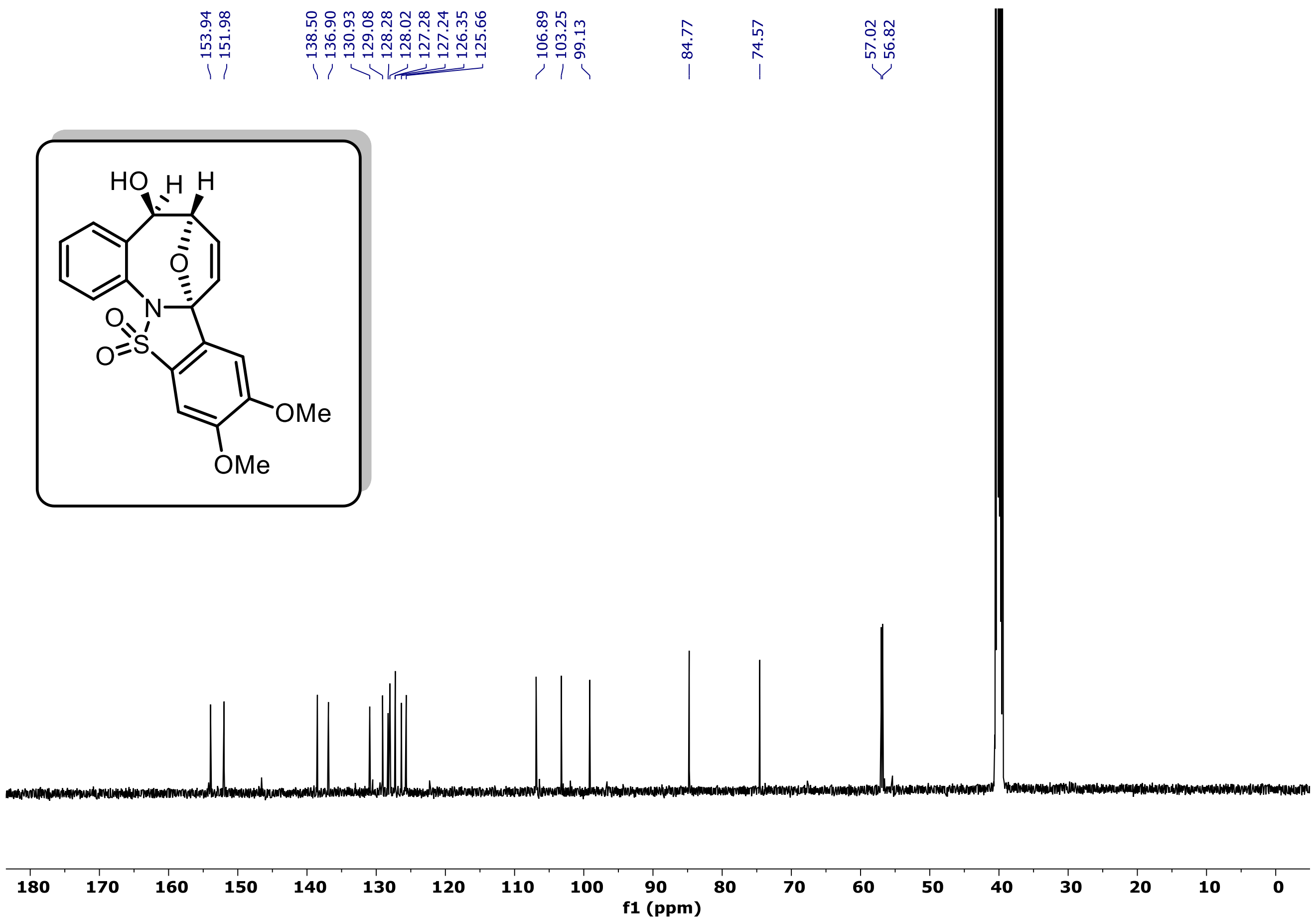

HSQC spectrum of **11c** (DMSO_d_6)

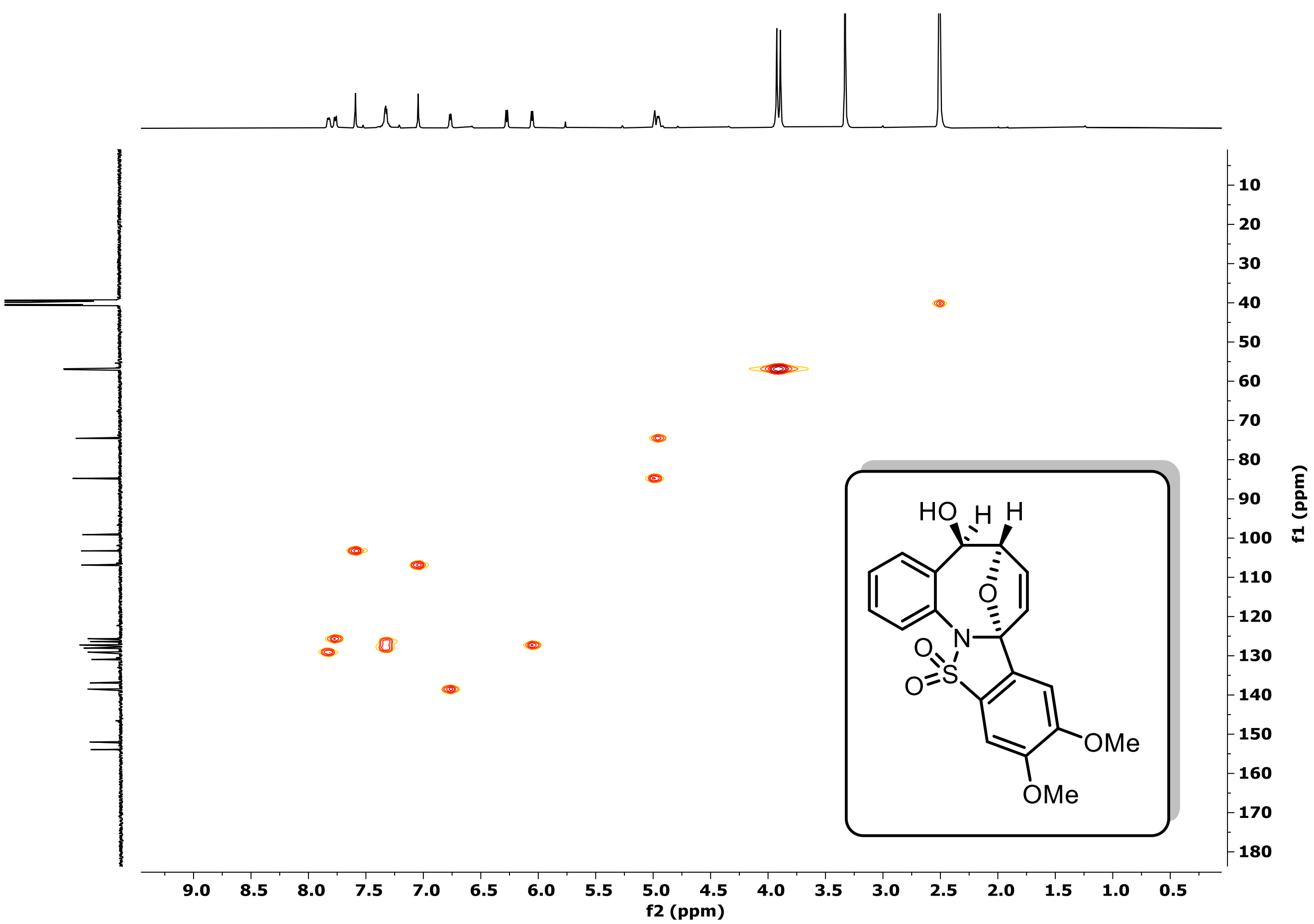

^1H NMR spectrum of **11d** (500 MHz, $CDCl_3$)

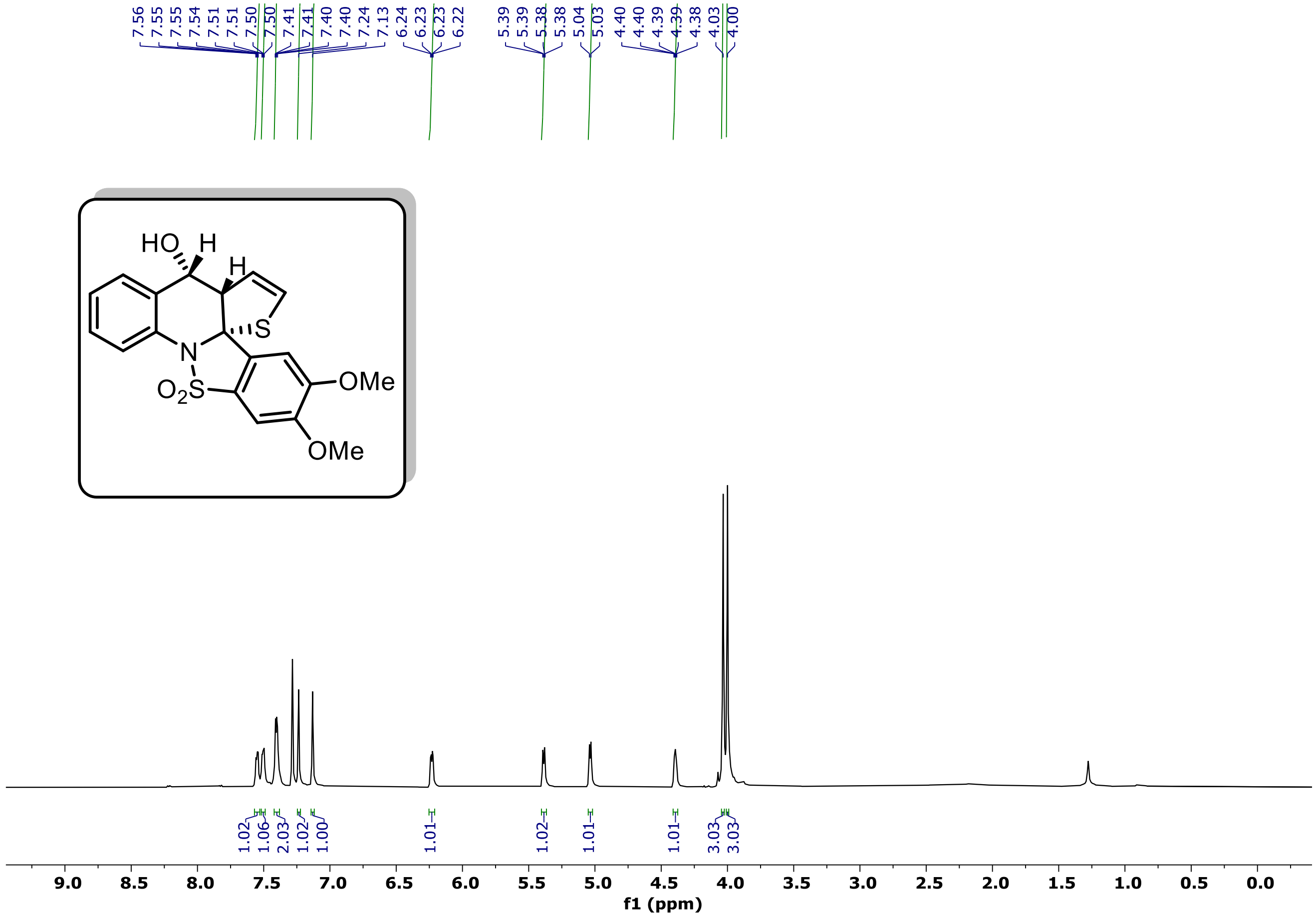

^{13}C NMR spectrum of **11d** (126 MHz, $CDCl_3$)

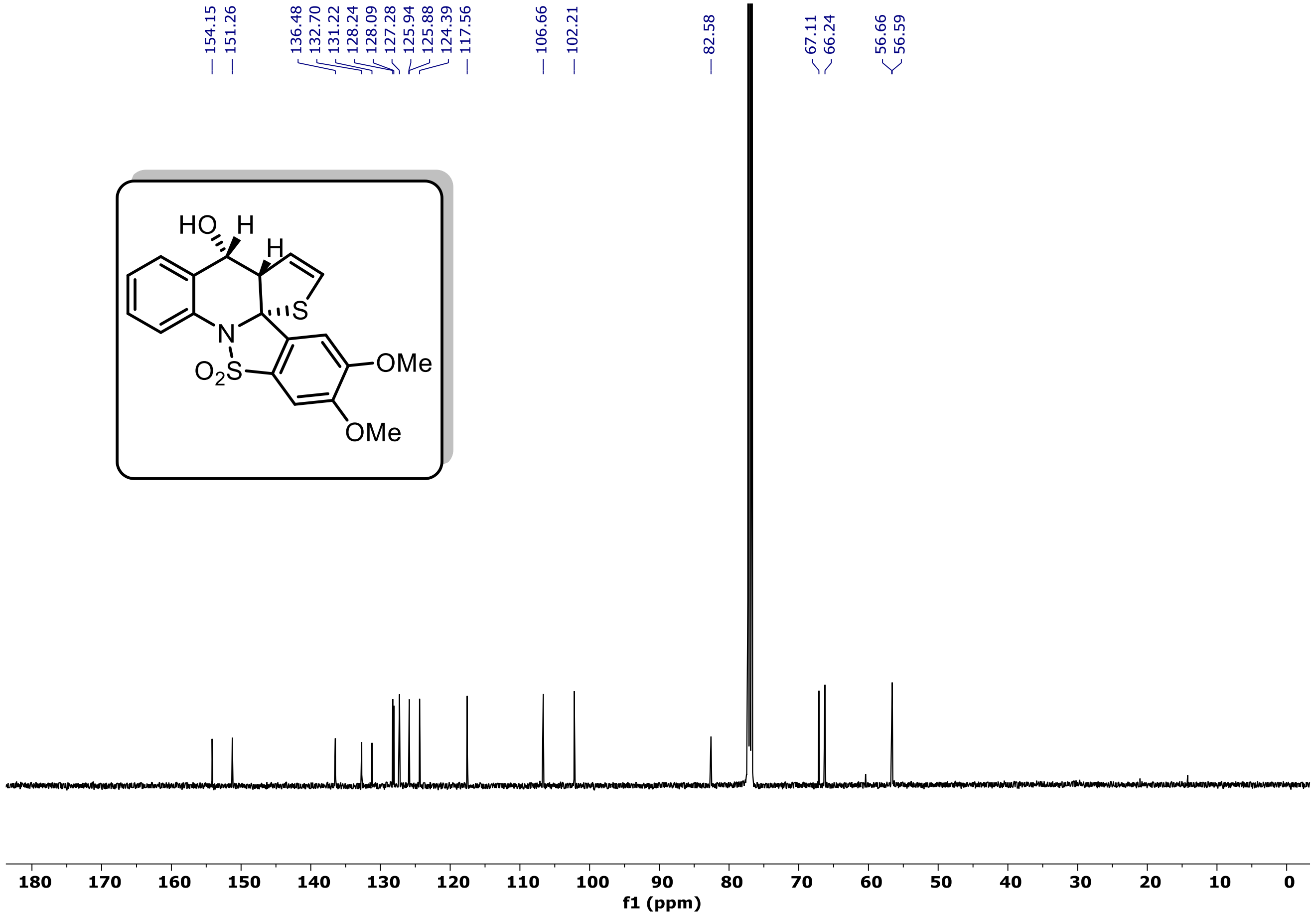

HSQC spectrum of **11d** ($CDCl_3$)

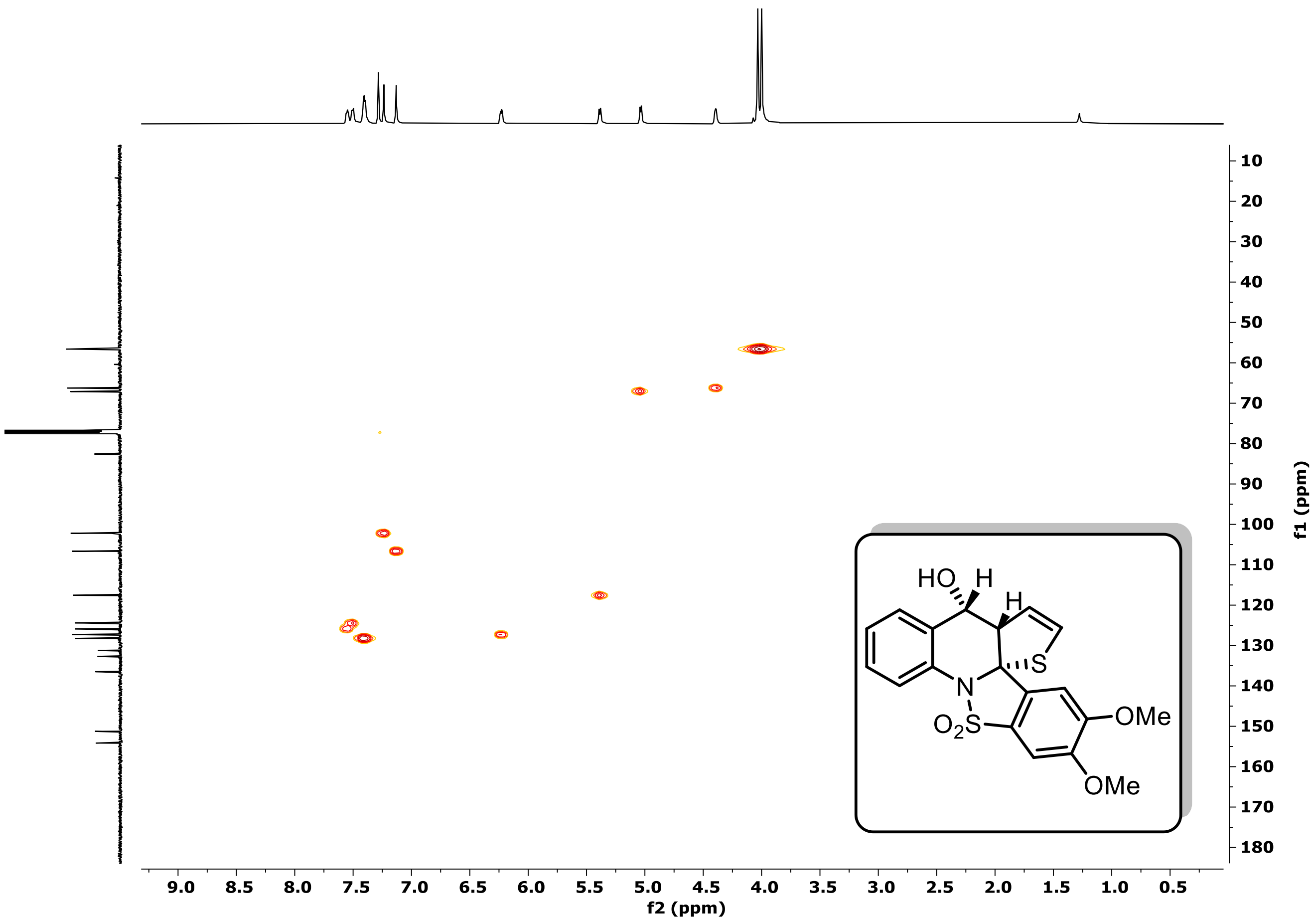

^{1}H NMR spectrum of **11e** (Major; 500 MHz, $CDCl_3$)

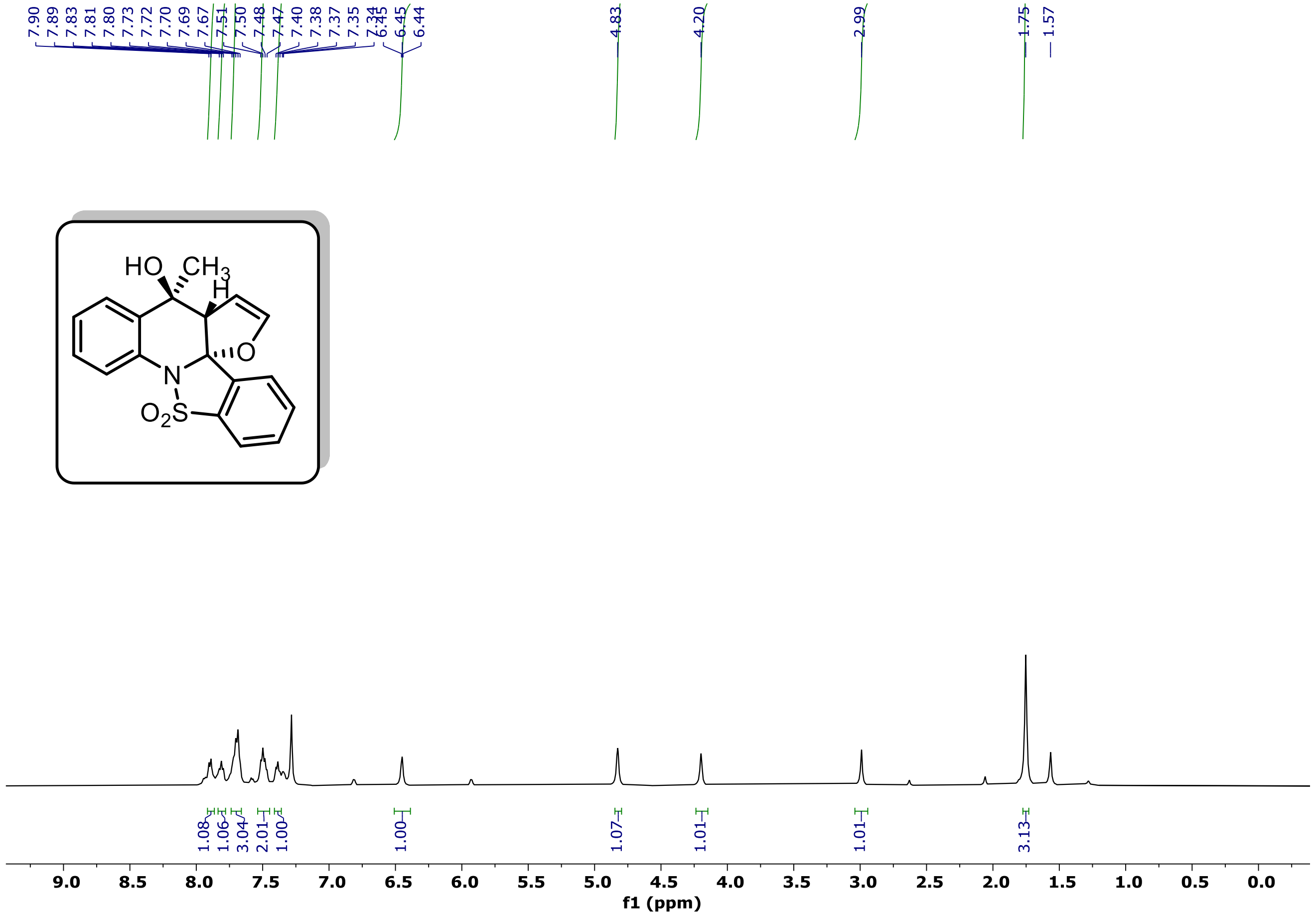

^{13}C NMR spectrum of **11e** (126 MHz, $CDCl_3$)

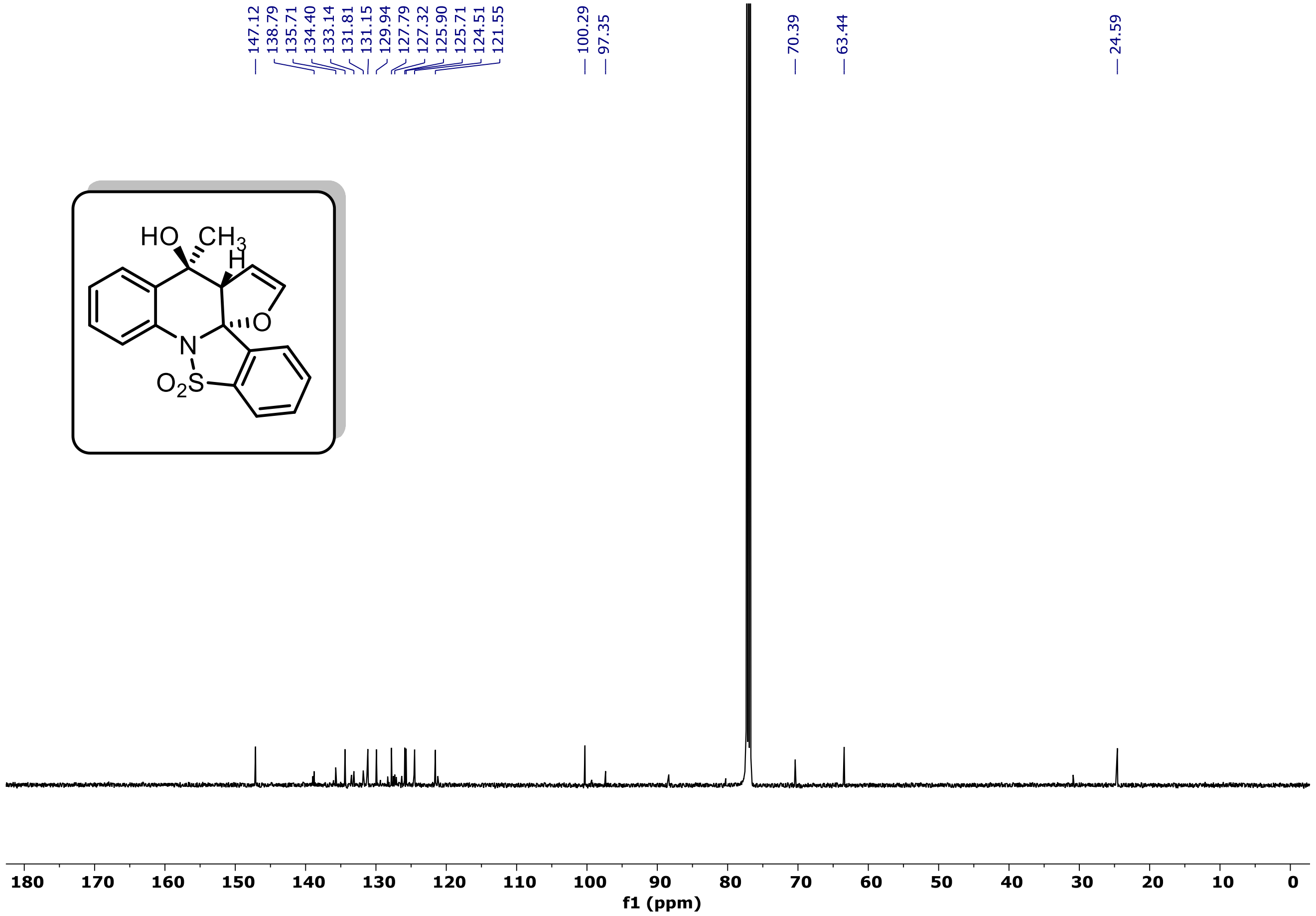

HSQC spectrum of **11e** ($CDCl_3$)

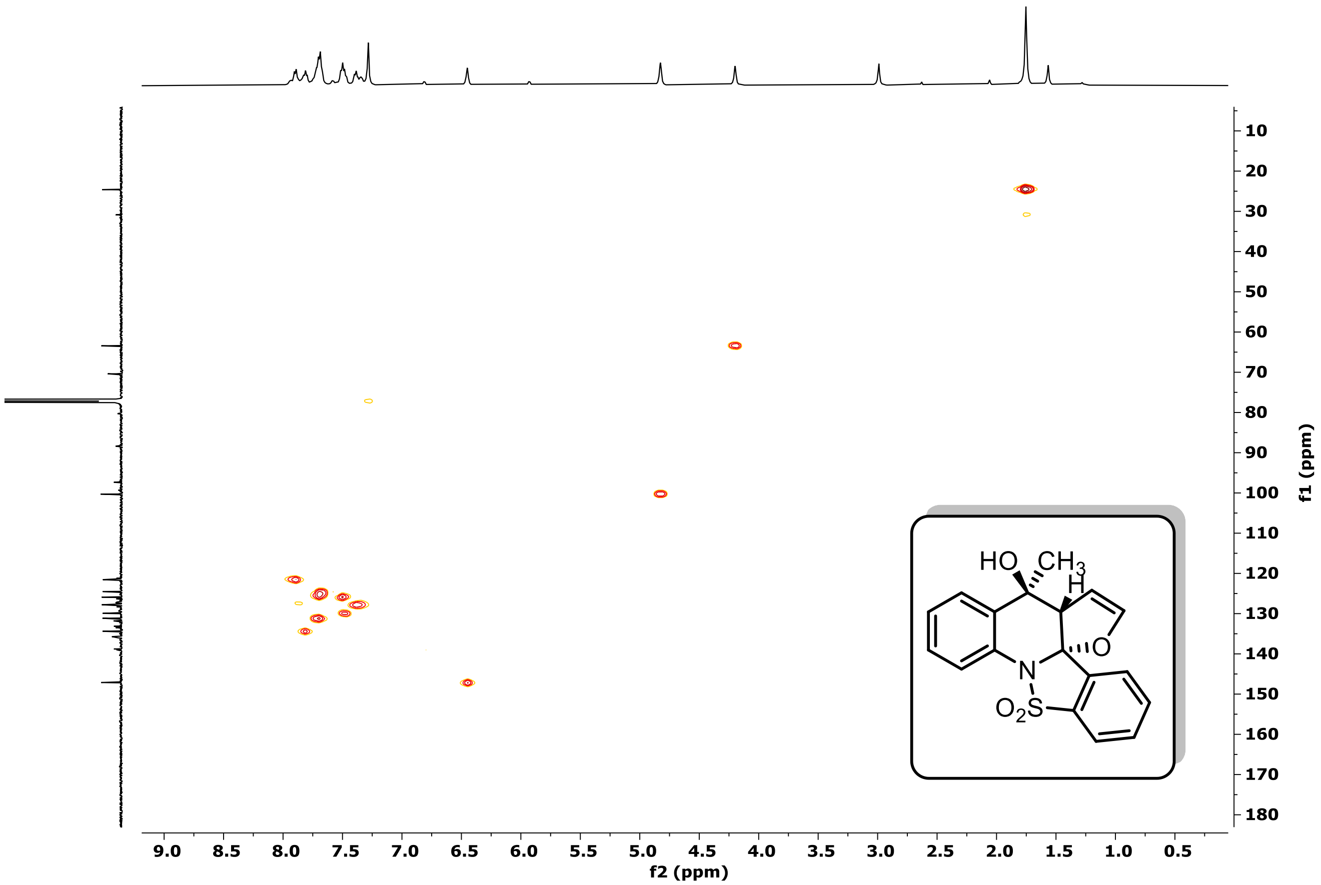

^{1}H NMR spectrum of **11ea** (500 MHz, $CDCl_3$)

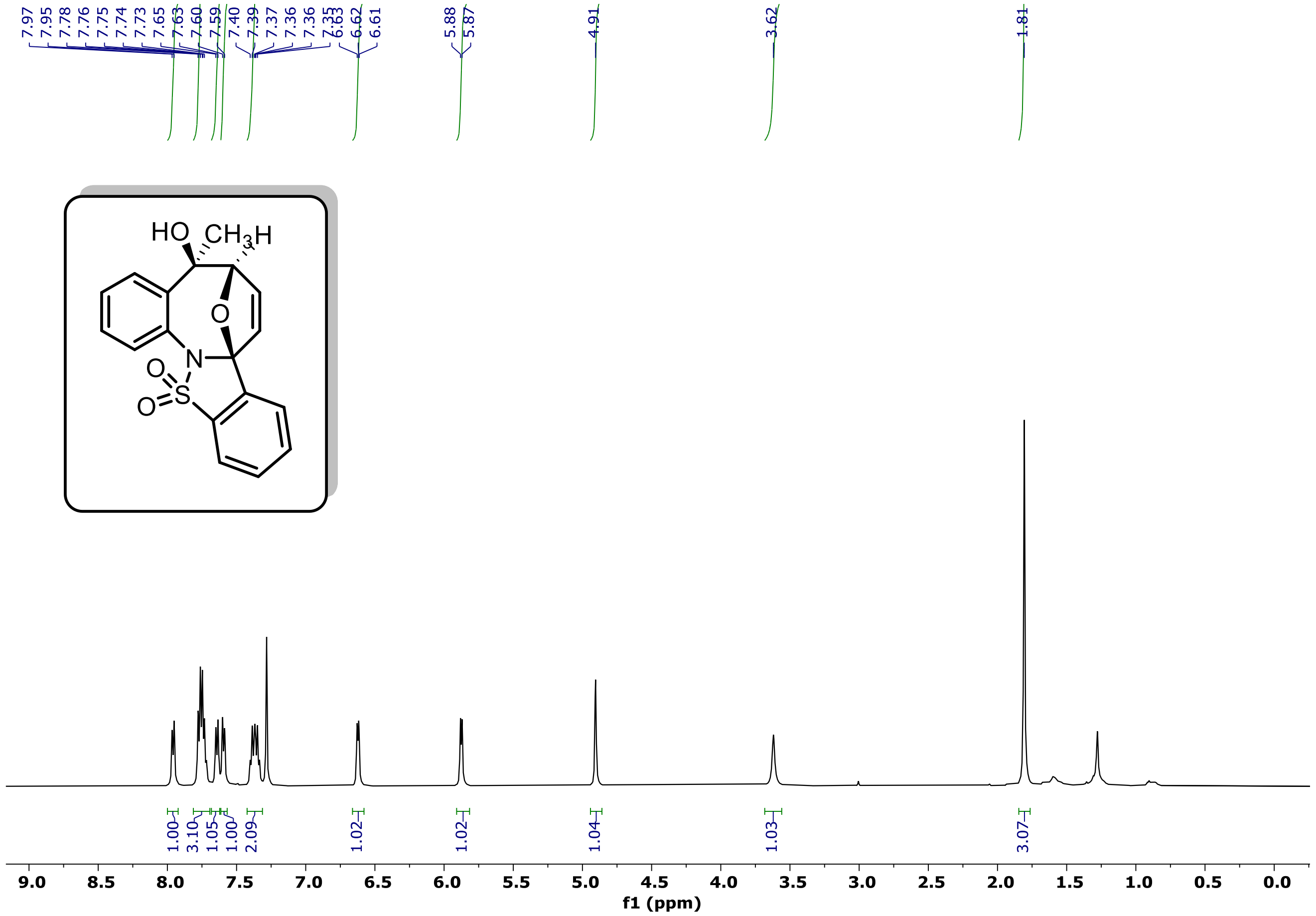

^{13}C NMR spectrum of **11ea** (126 MHz, $CDCl_3$)

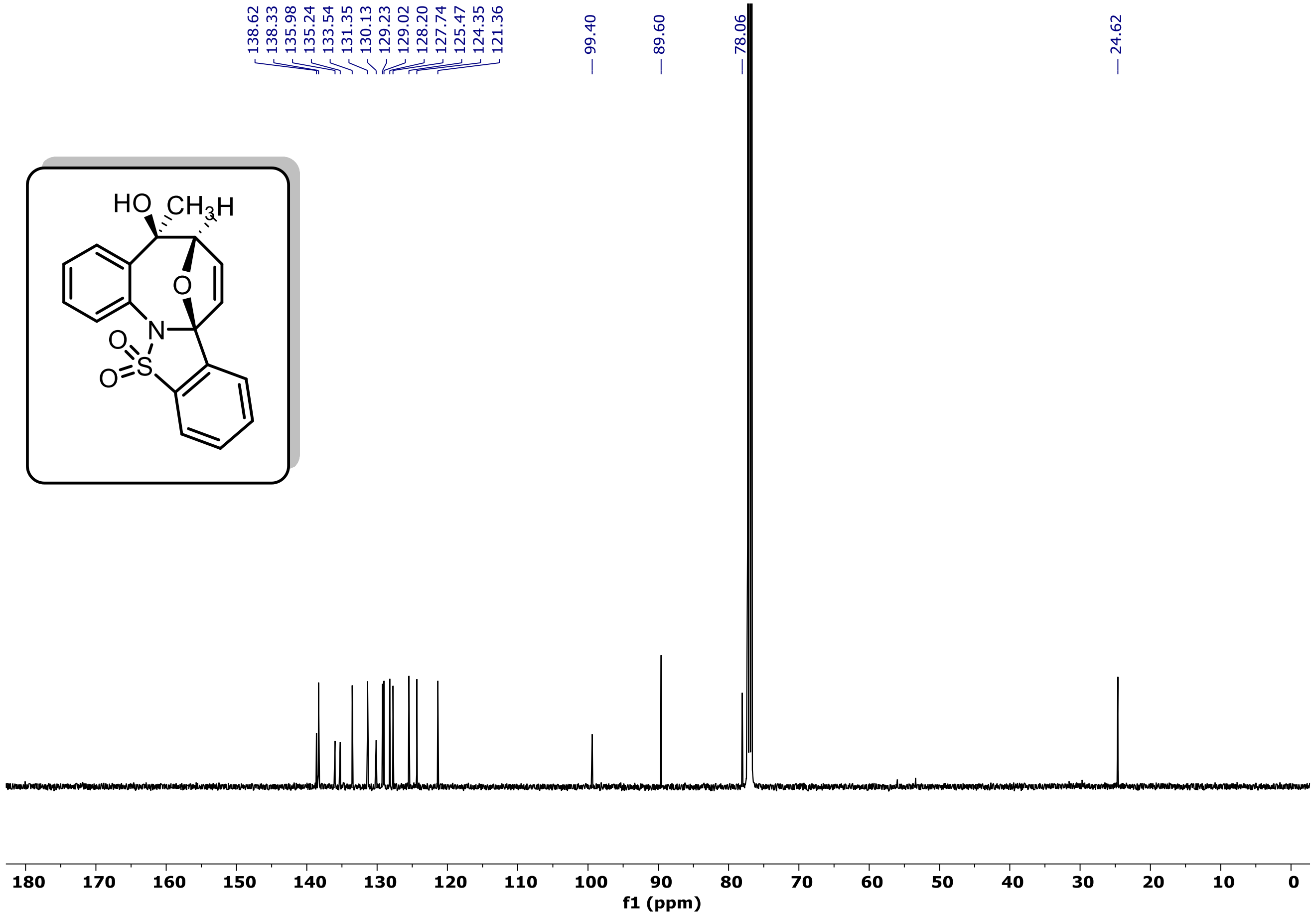

HSQC spectrum of **11ea** ($CDCl_3$)

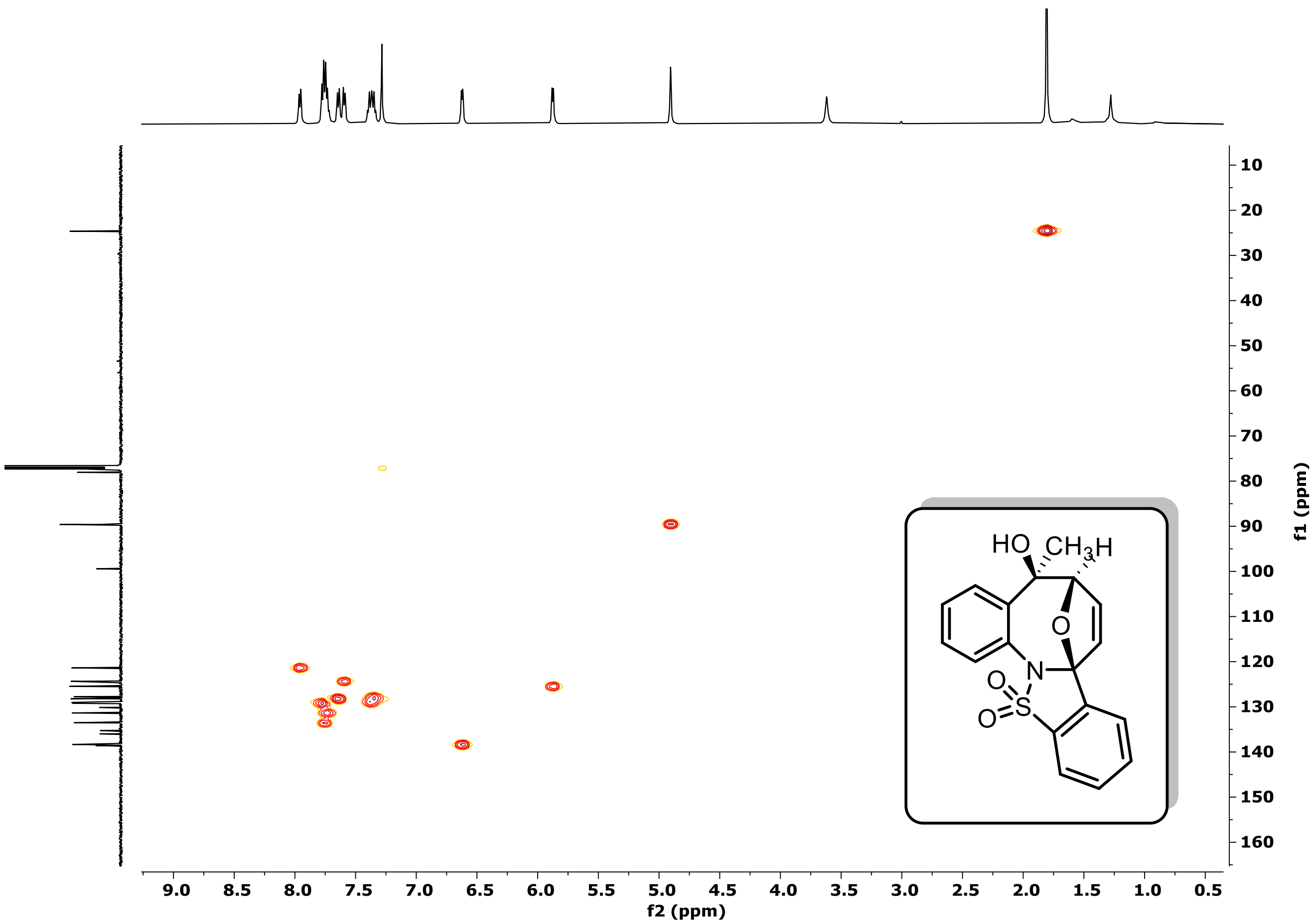

^{1}H NMR spectrum of **11f** (500 MHz, $CDCl_3$)

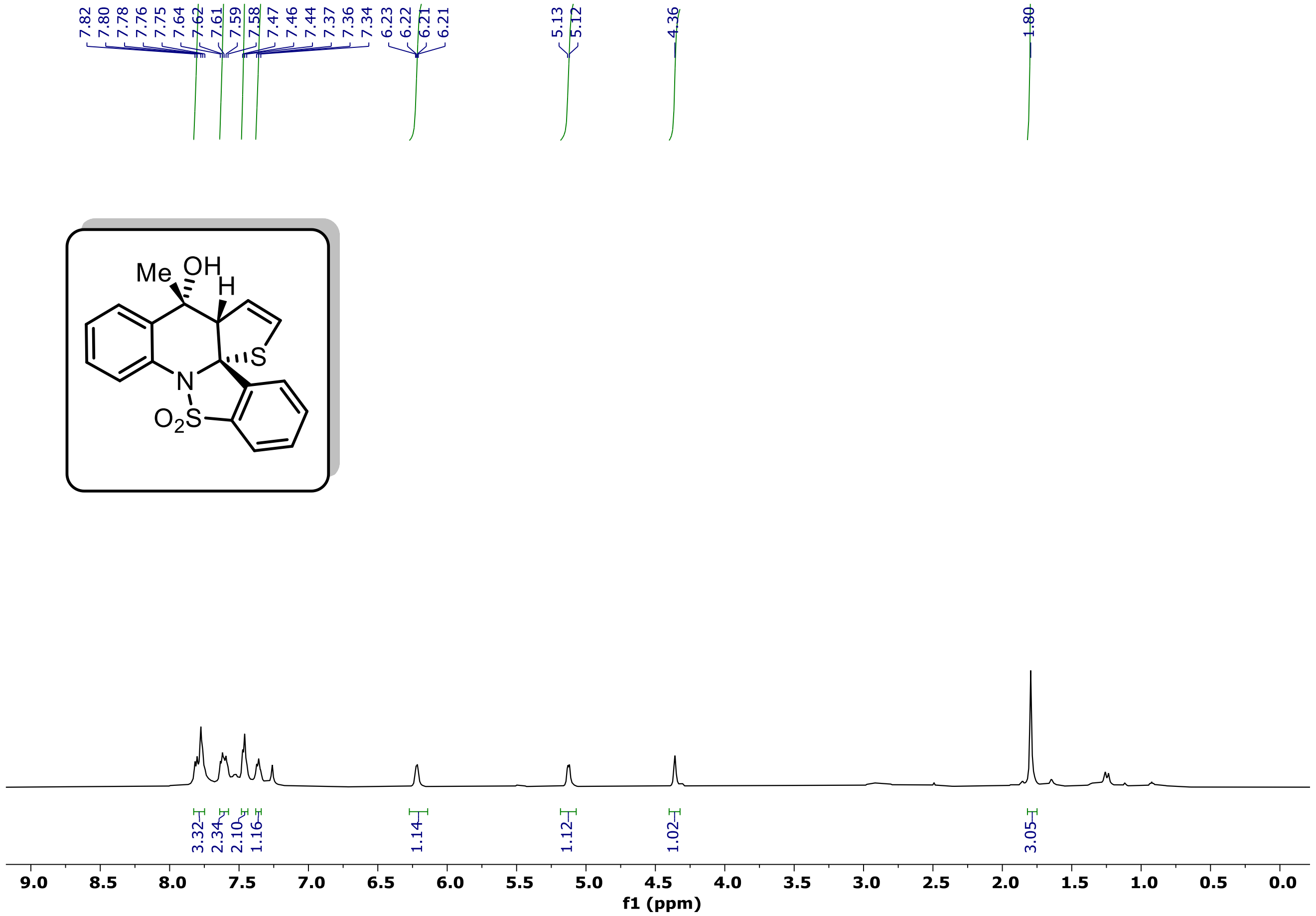

^{13}C NMR spectrum of **11f** (126 MHz, $CDCl_3$)

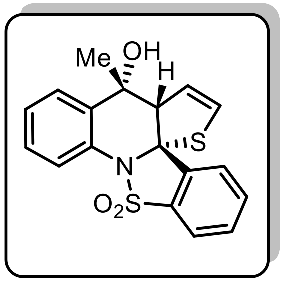

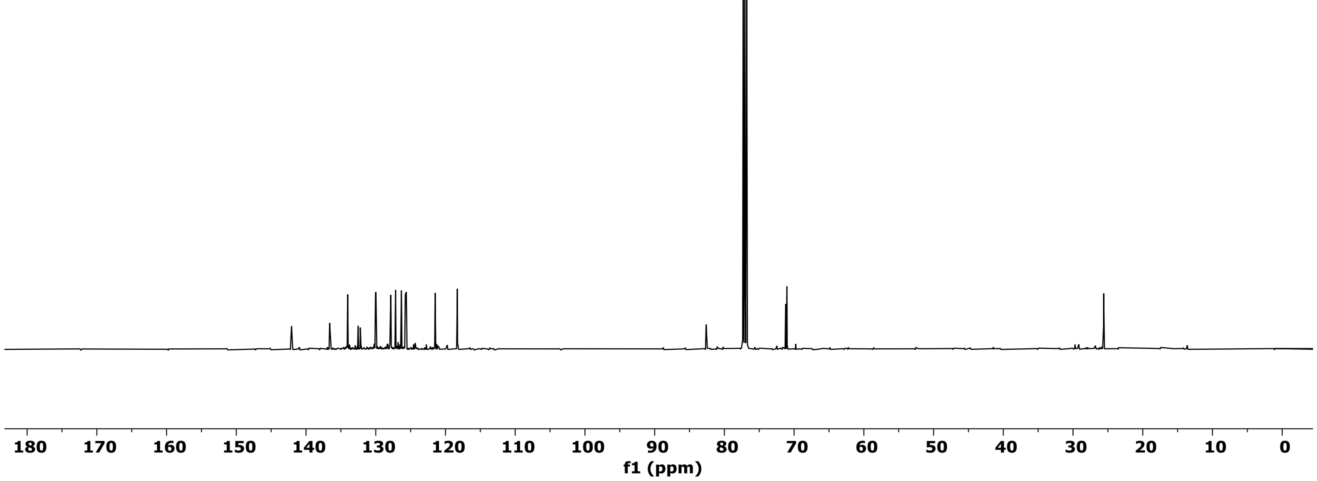

HSQC spectrum of **11f** ($CDCl_3$)

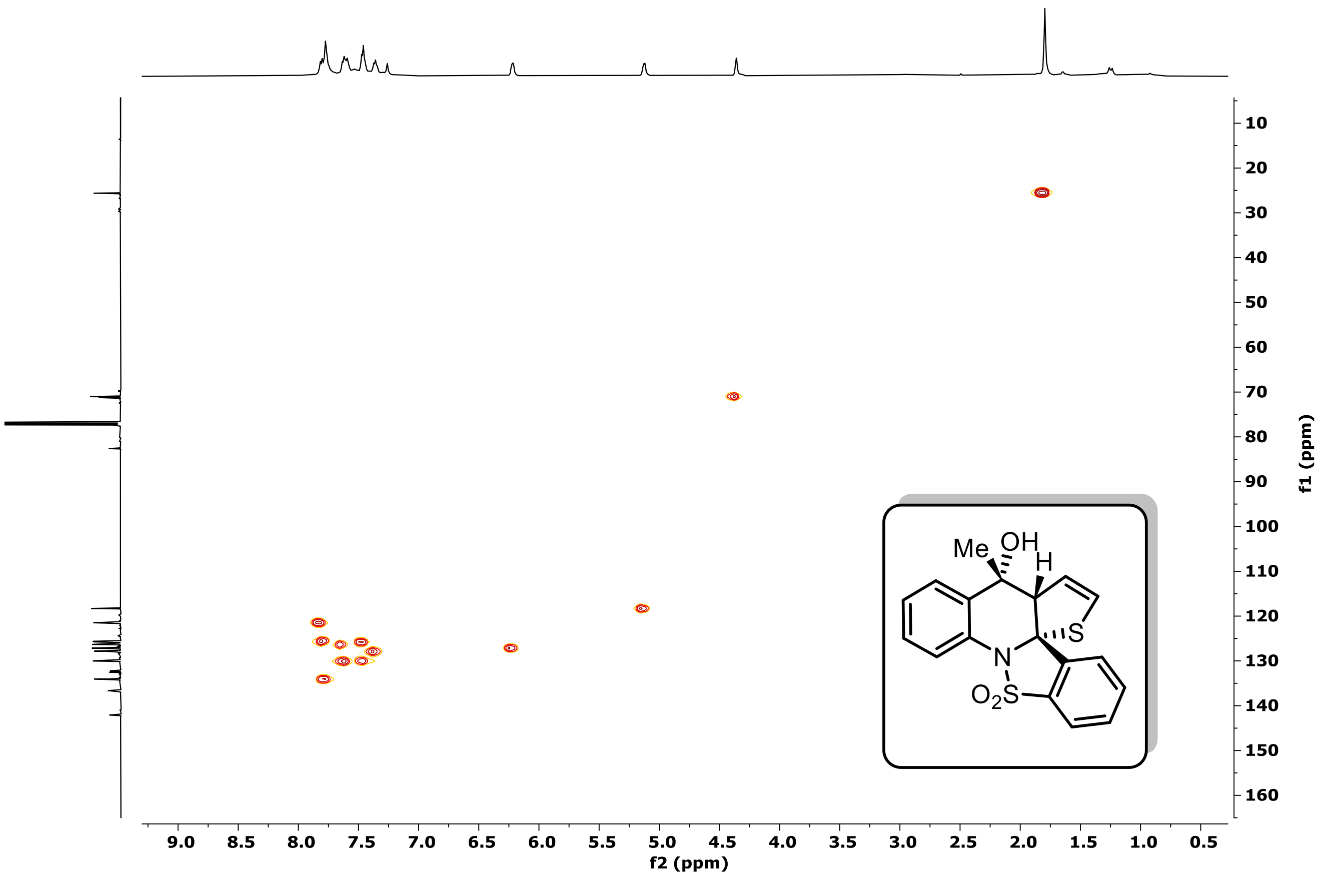

^{1}H NMR spectrum of **11g** (500 MHz, $CDCl_3$)

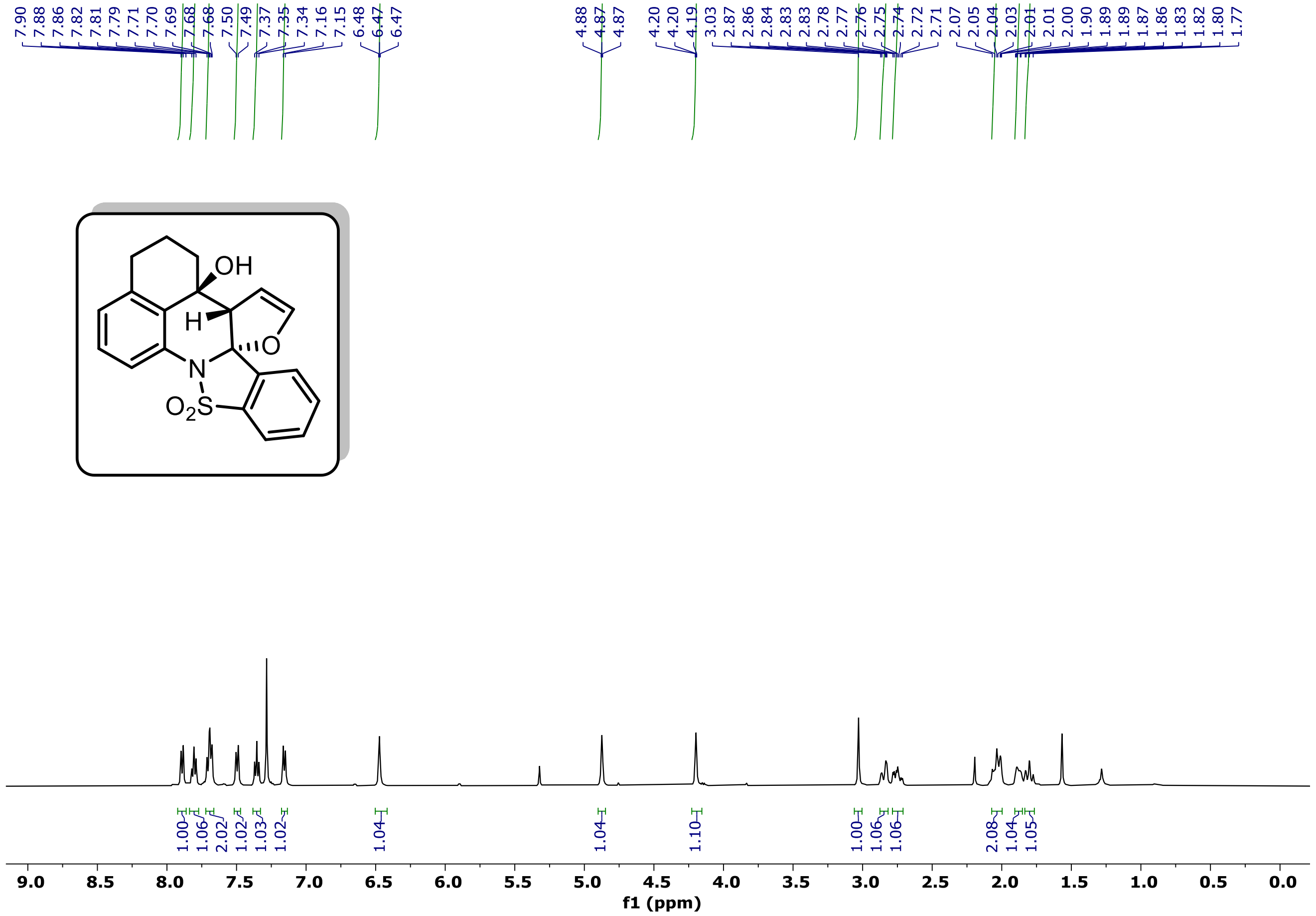

^{13}C NMR spectrum of **11g** (126 MHz, $CDCl_3$)

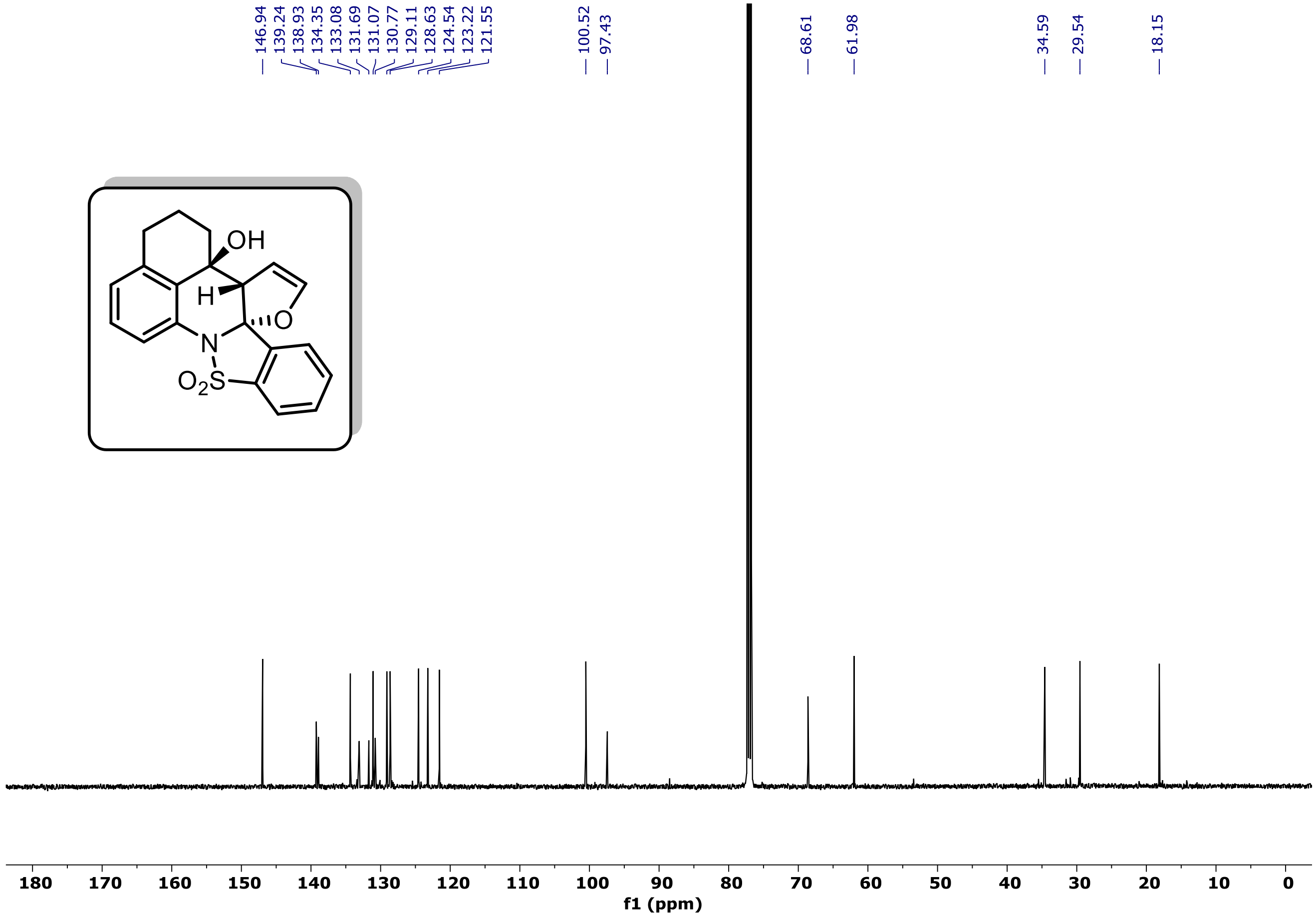

HSQC spectrum of **11g** ($CDCl_3$)

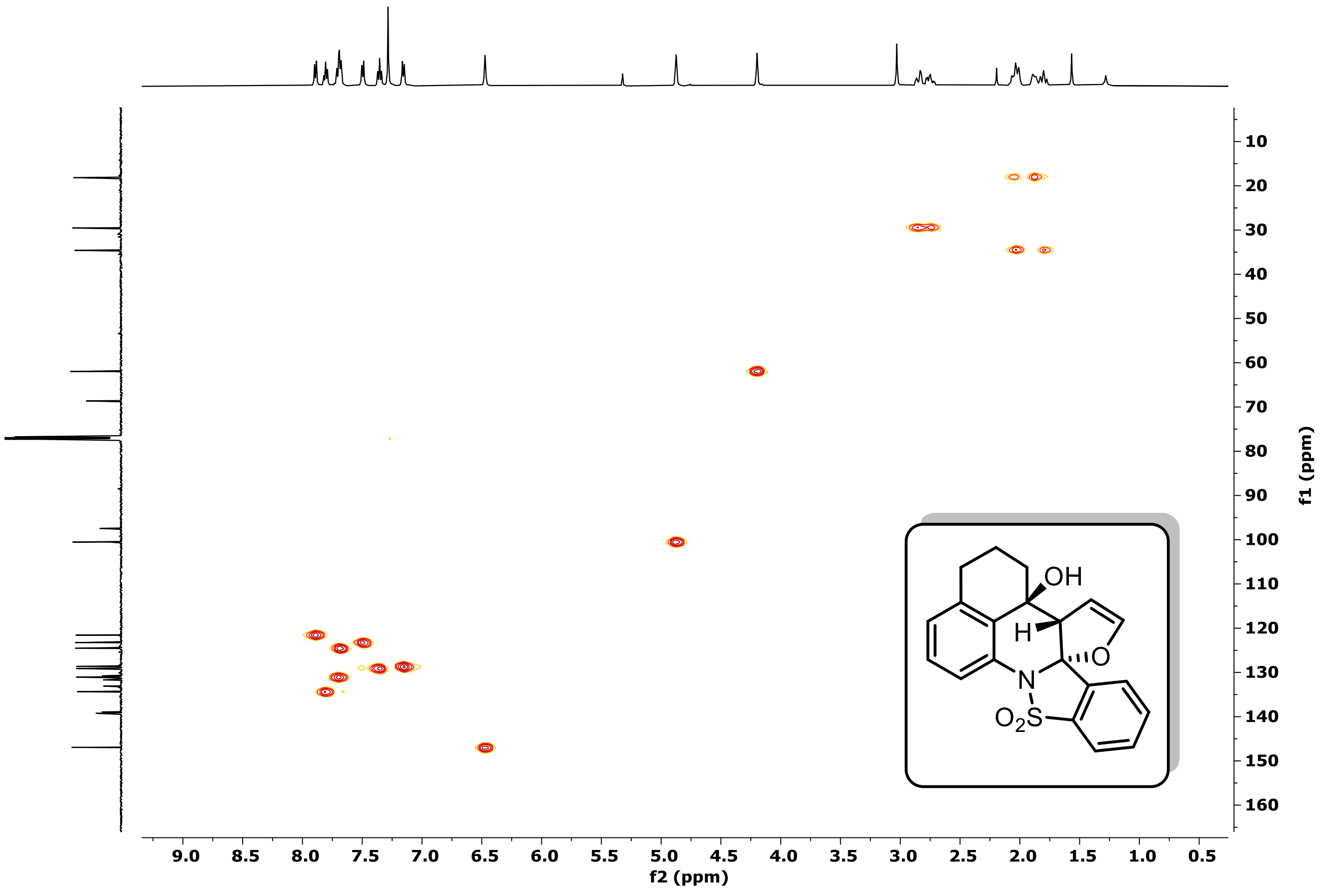

^{1}H NMR spectrum of **12** (500 MHz, $CDCl_3$)

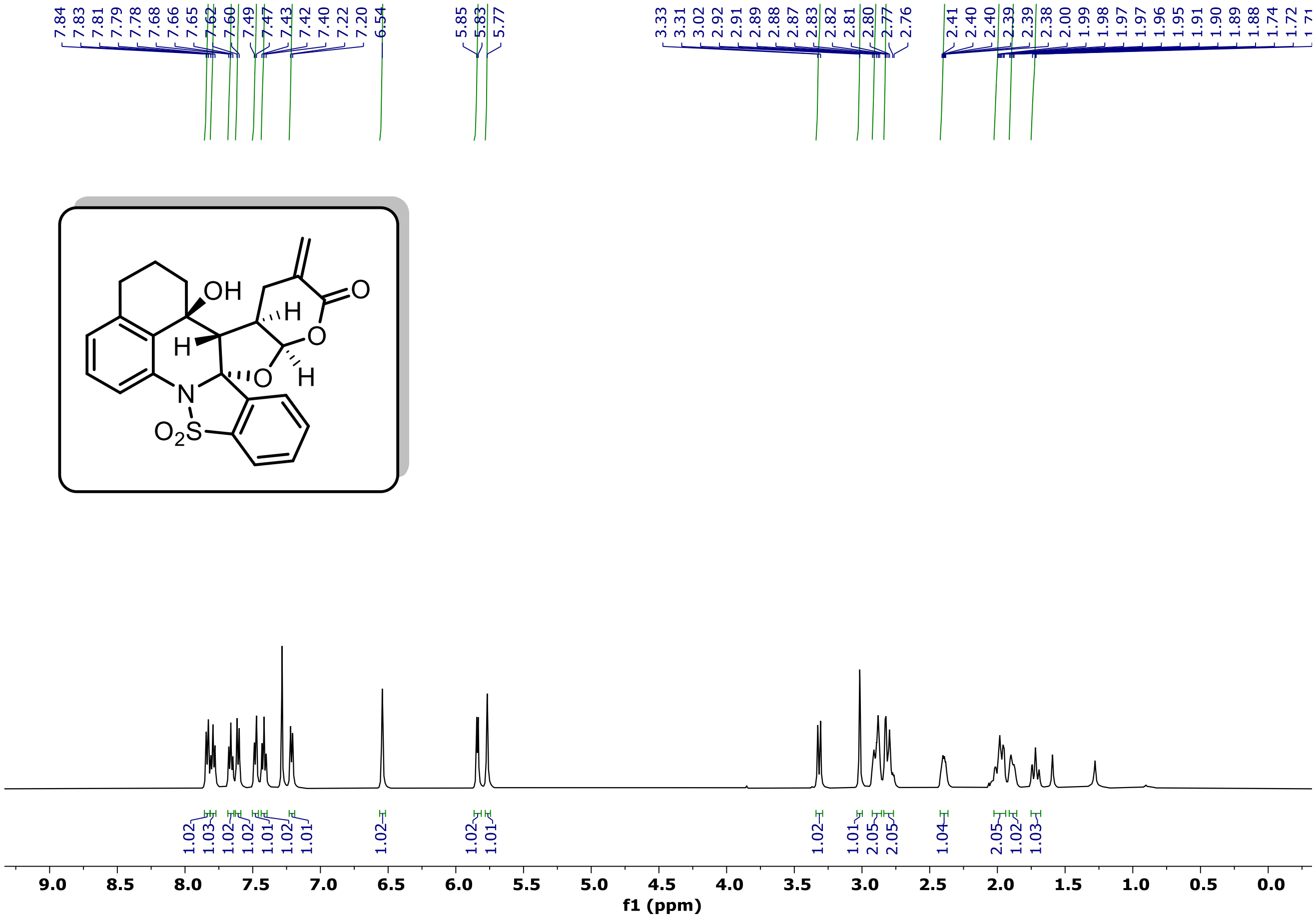

^{13}C NMR spectrum of **12** (126 MHz, $CDCl_3$)

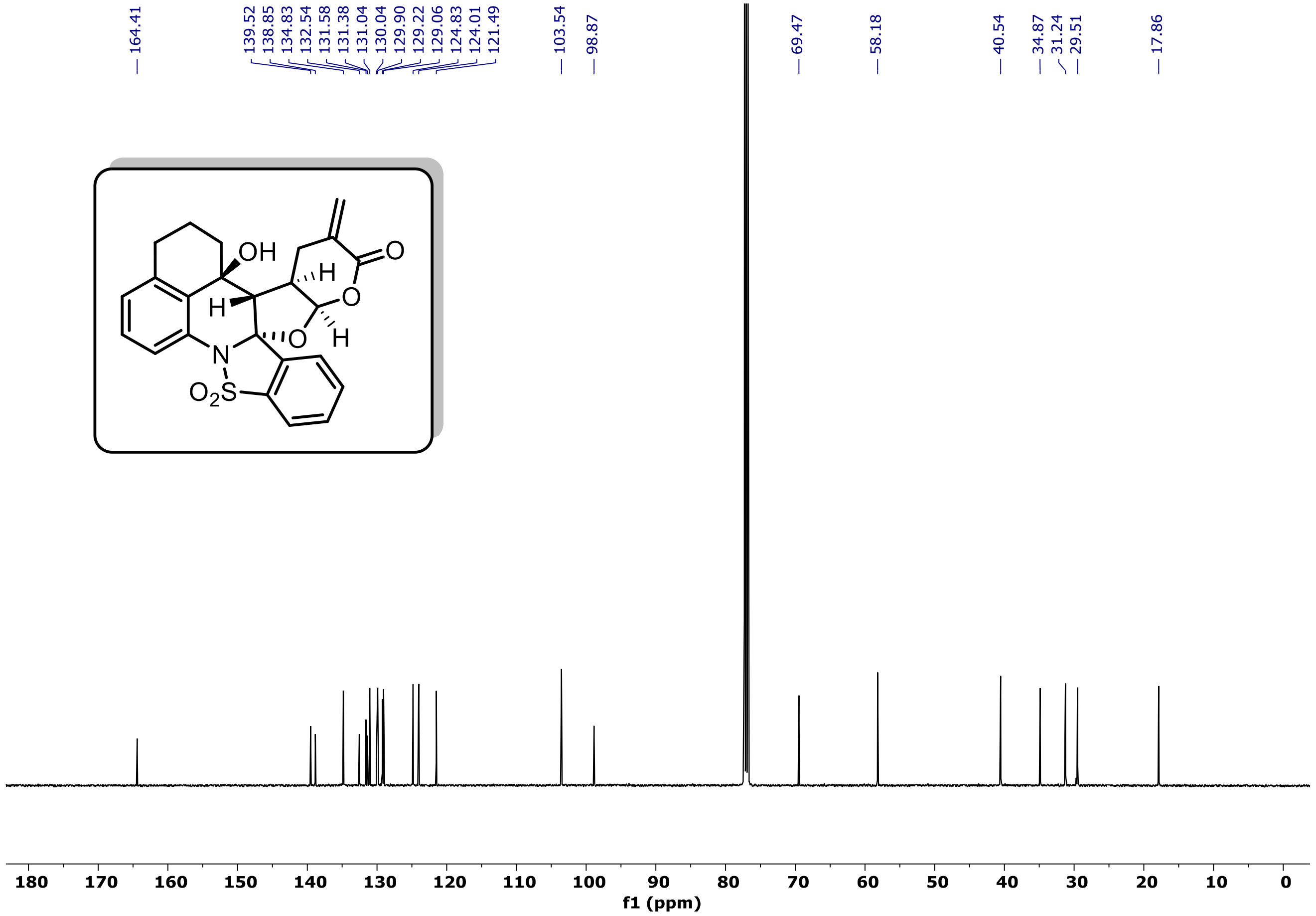

HSQC spectrum of **12** ($CDCl_3$)

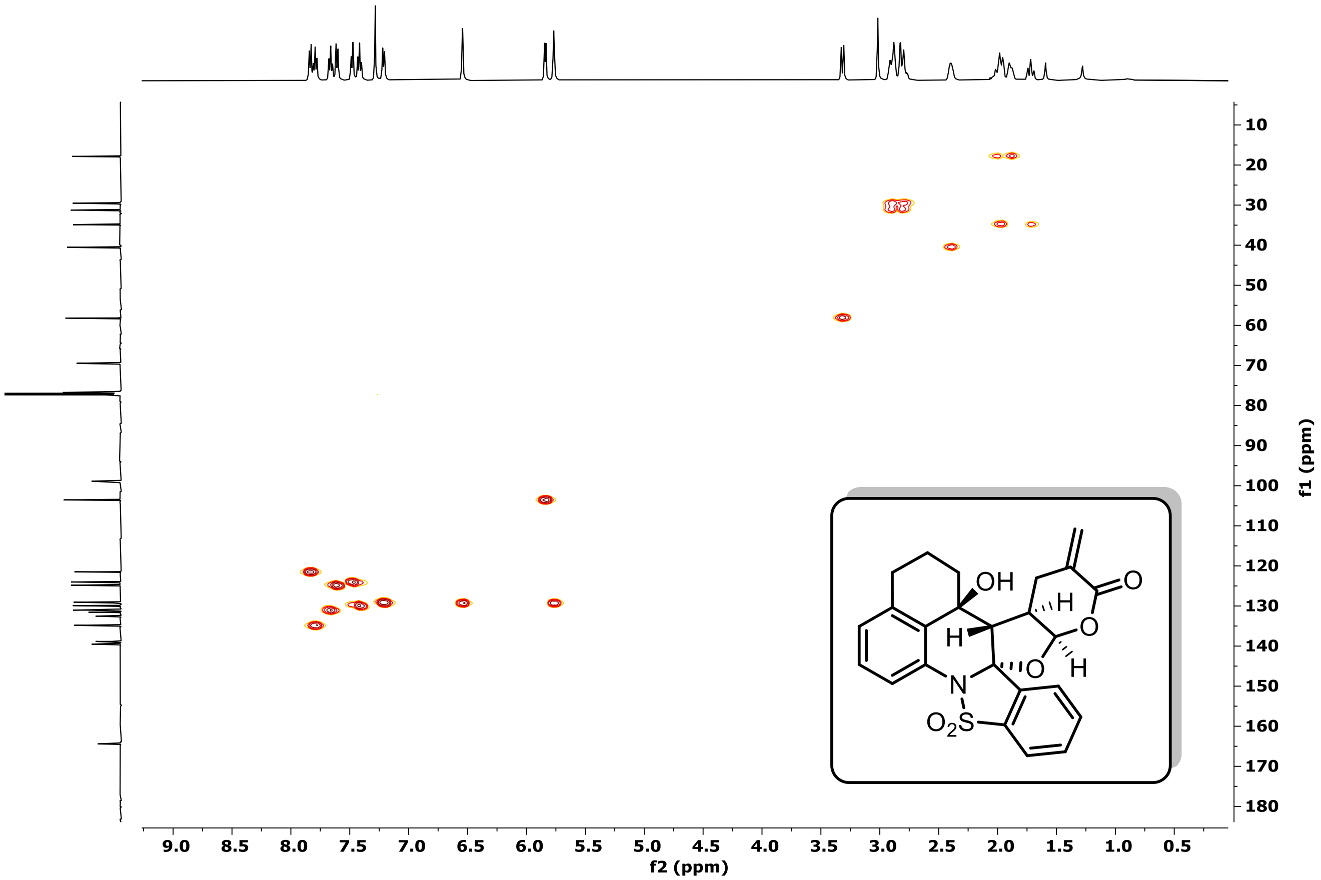

^{1}H NMR spectrum of **13** (500 MHz, $CDCl_3$)

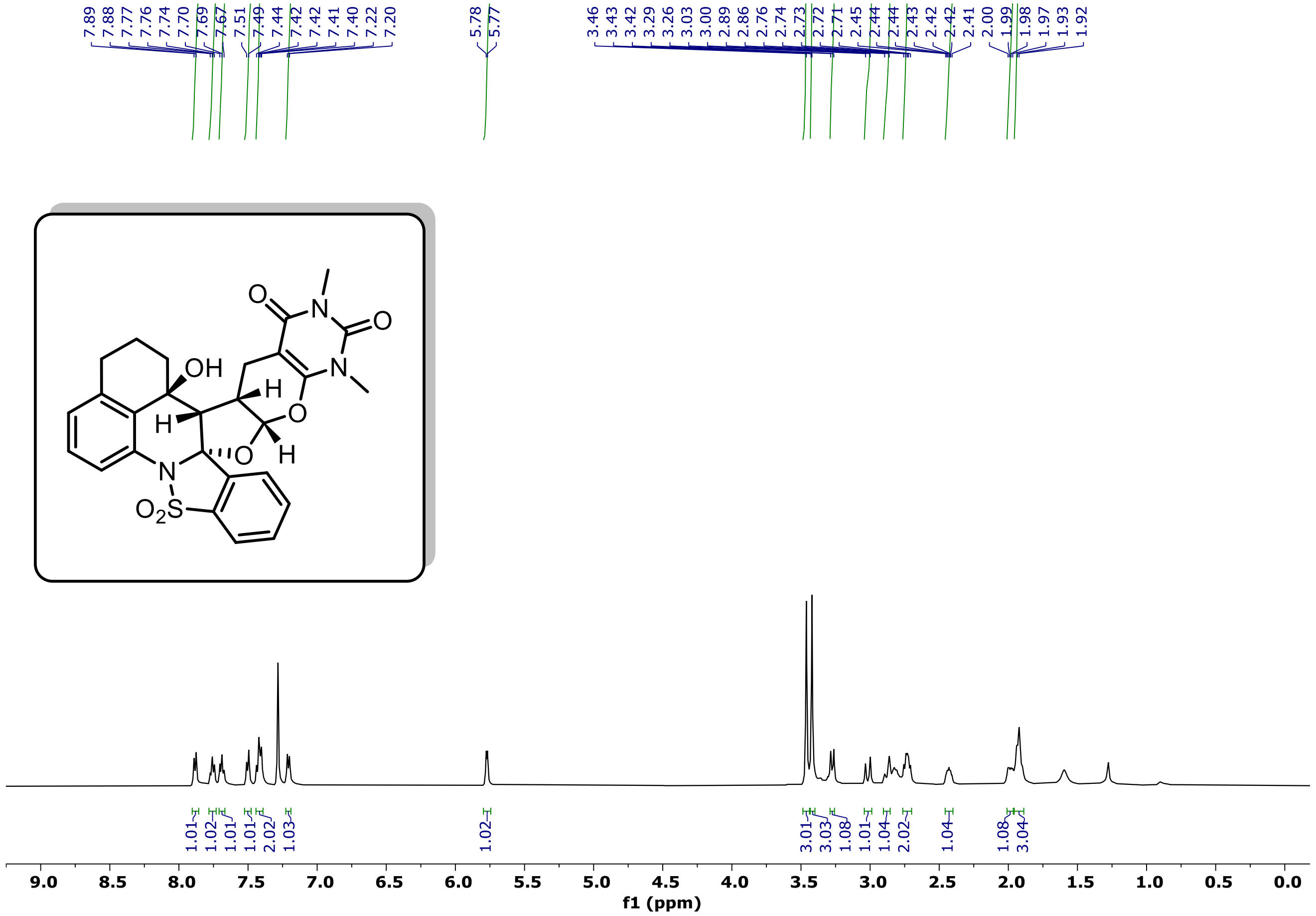

^{13}C NMR spectrum of **13** (126 MHz, $CDCl_3$)

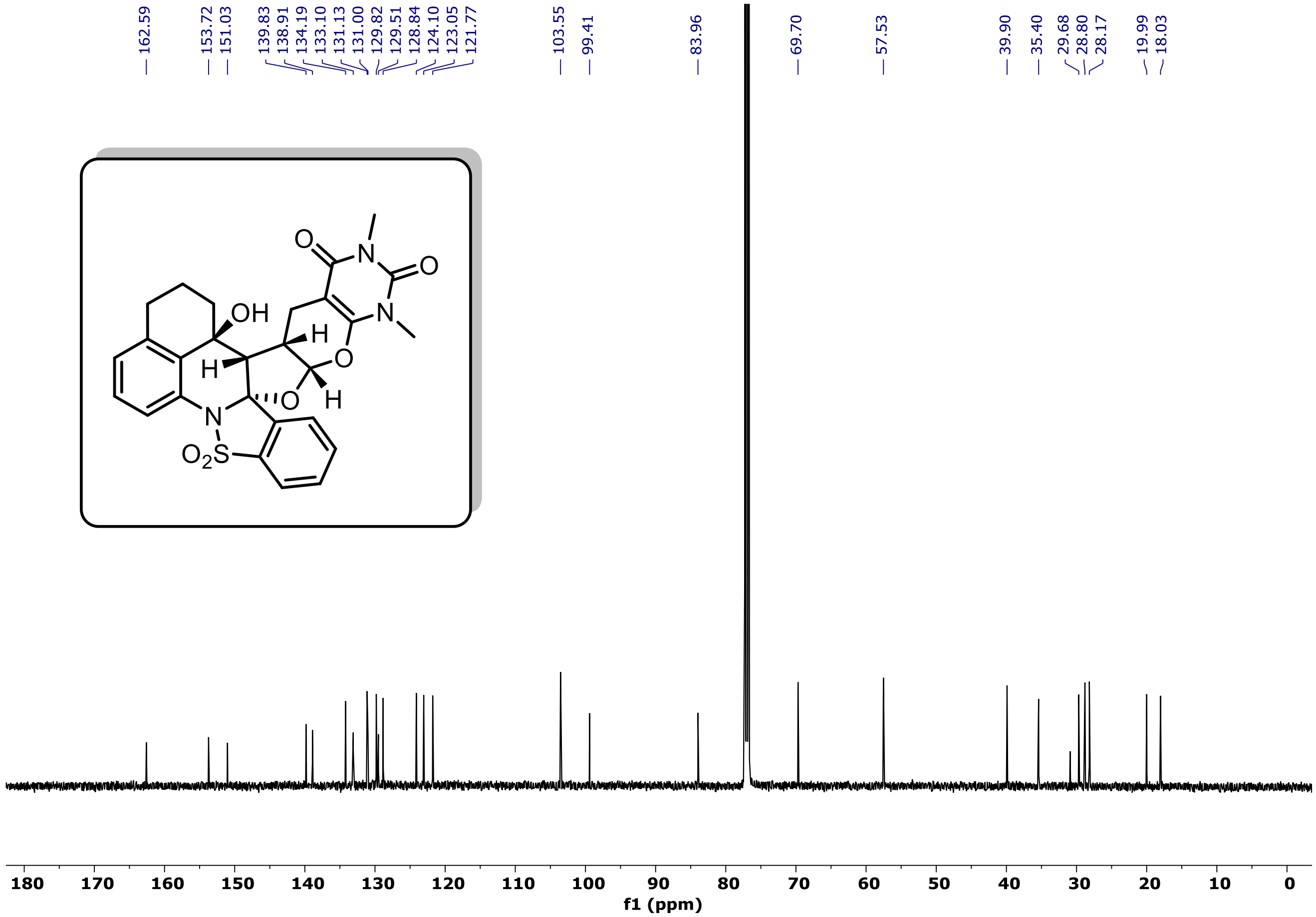

HSQC spectrum of **13** ($CDCl_3$)

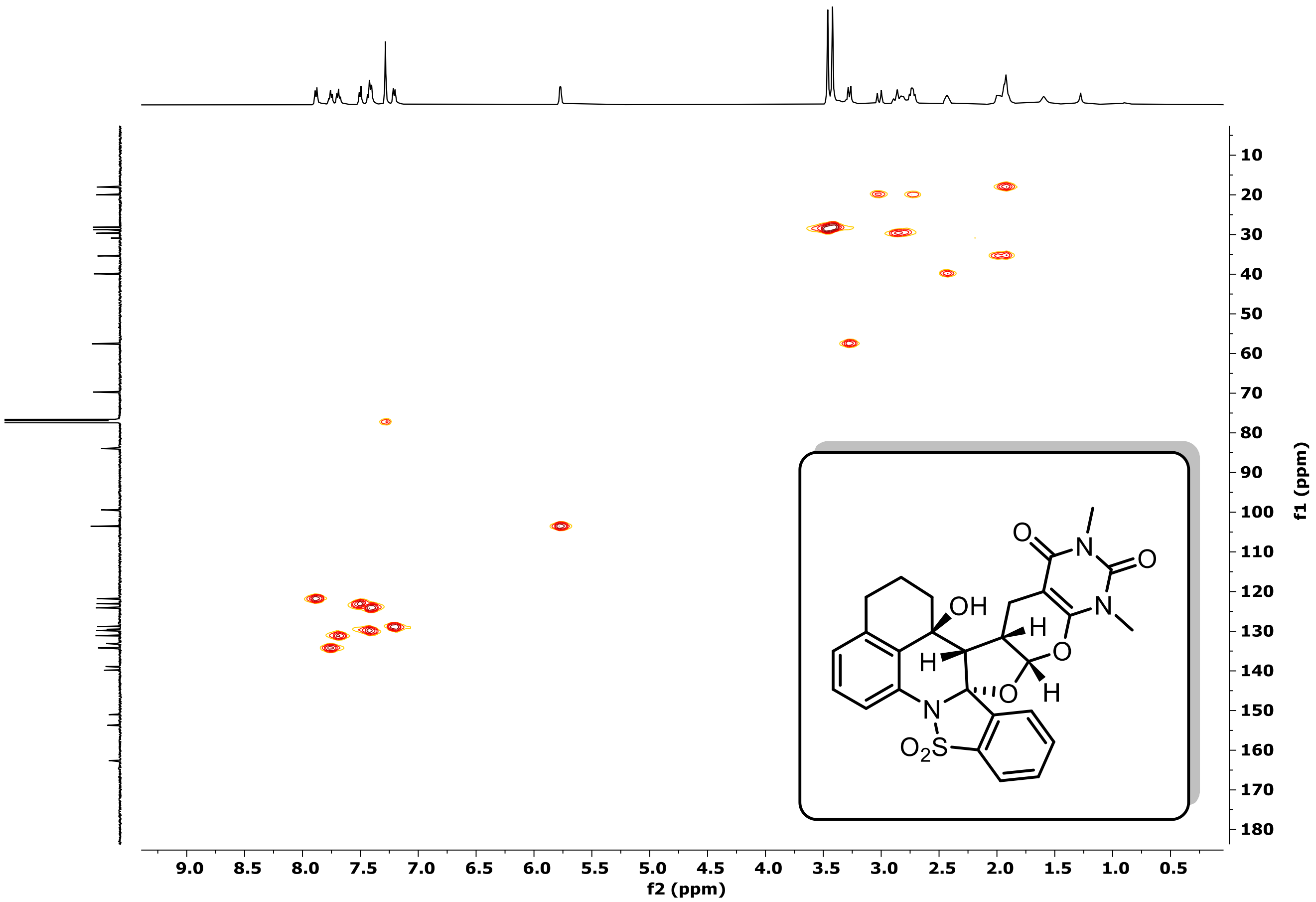

NOESY spectrum of **13** ($CDCl_3$)

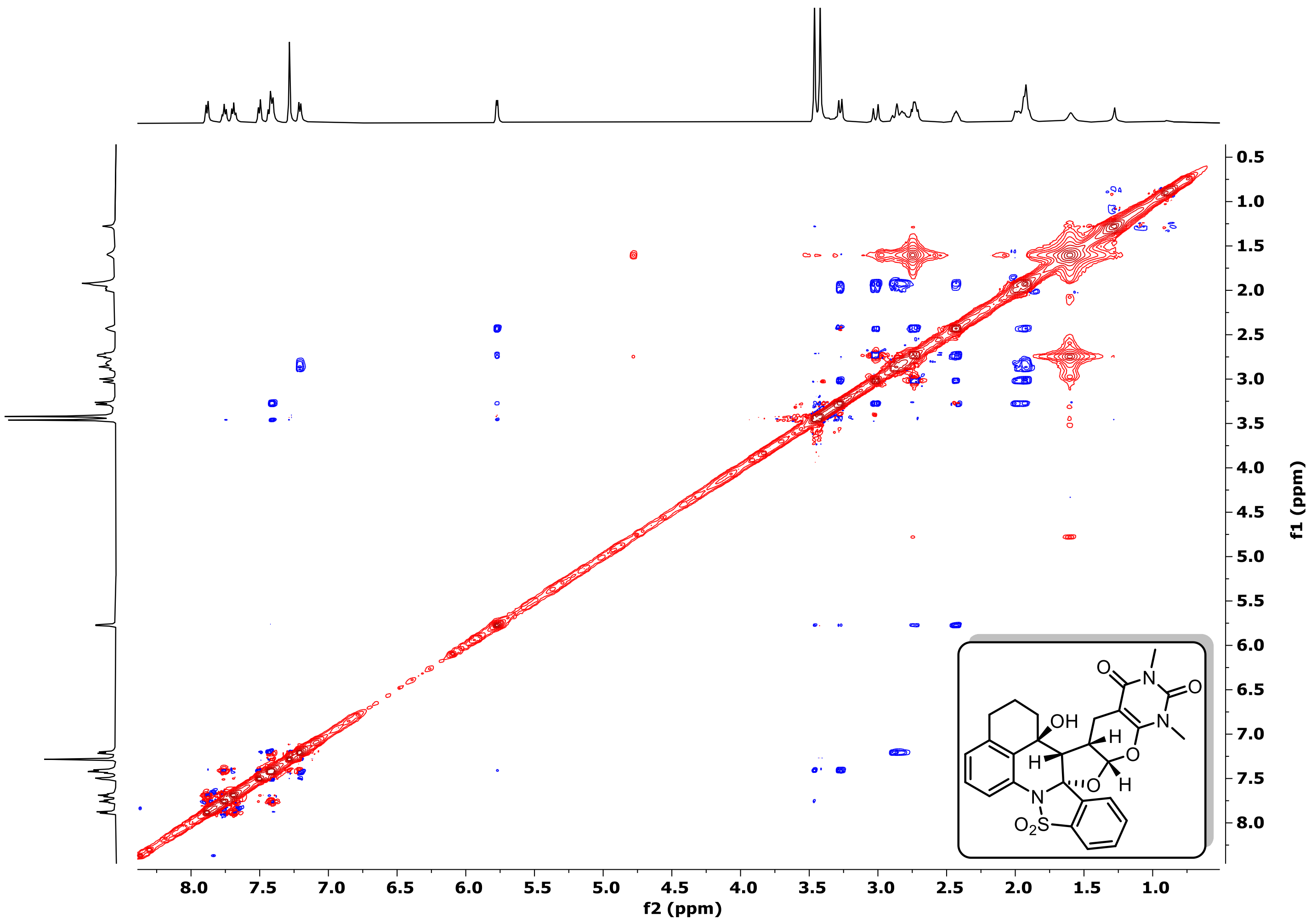

UV Spectra of photoprecursors

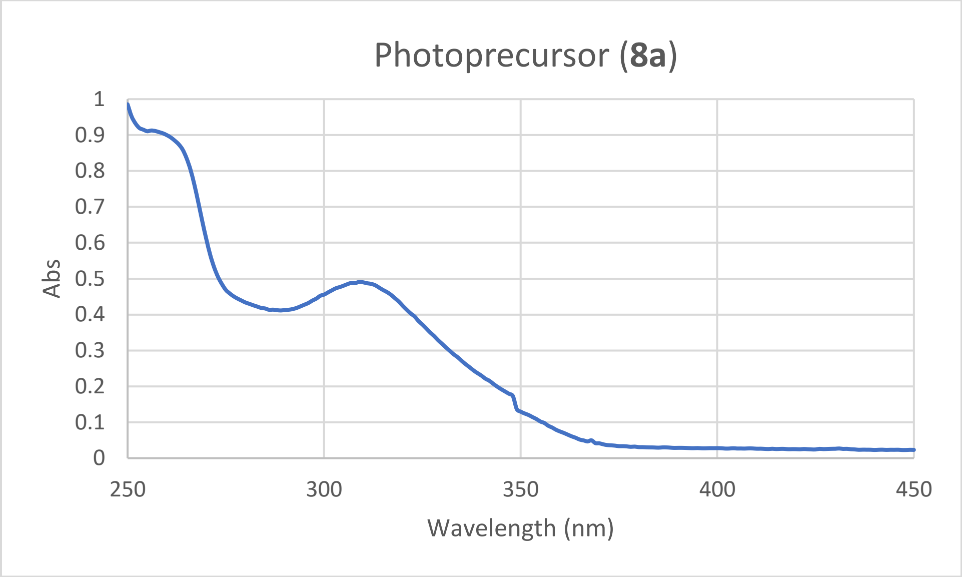

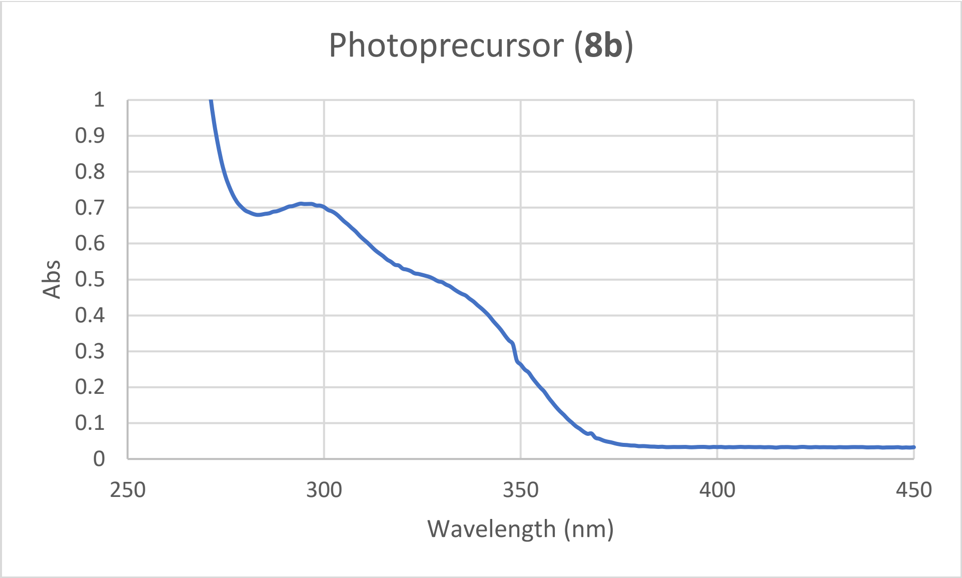

Photoprecursor (**8b**)
Abs
1
0.9
0.8
0.7
0.6
0.5
0.4
0.3
0.2
0.1
0
250
300
350
400
450
Wavelength (nm)

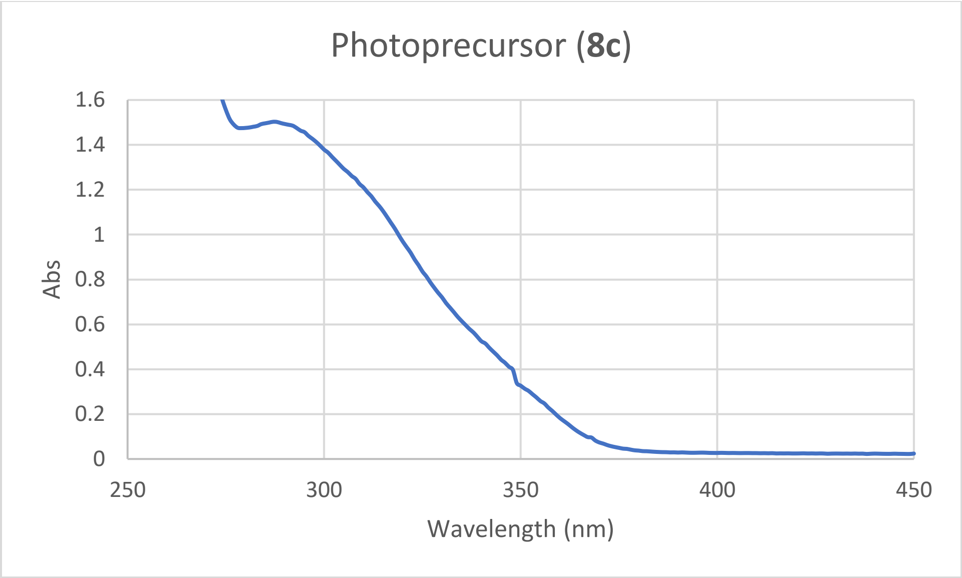
Photoprecursor (**8c**)
1.6
1.4
1.2
1
0.8
0.6
0.4
0.2
0
Abs
250
300
350
400
450
Wavelength (nm)

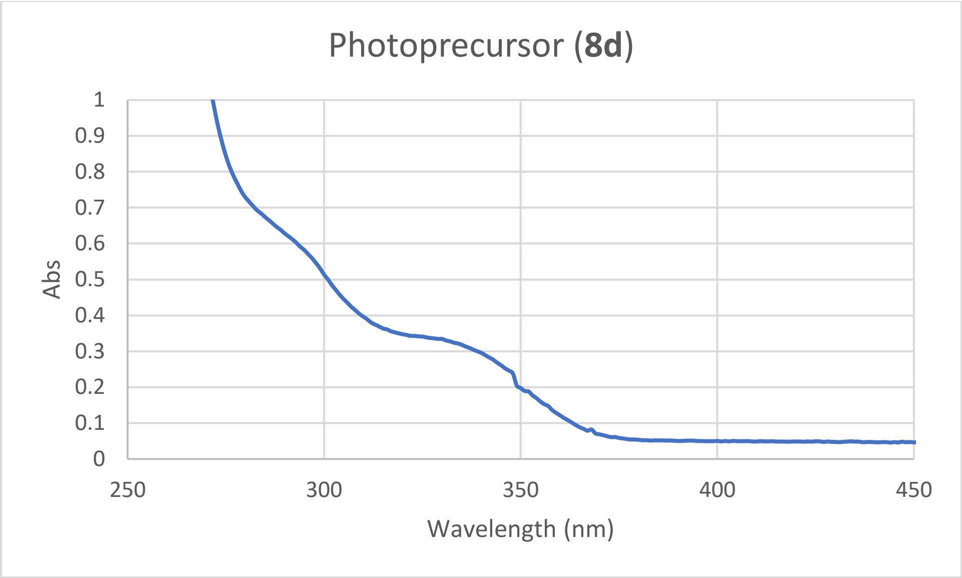
Photoprecursor (**8d**)
1
0.9
0.8
0.7
0.6
0.5
0.4
0.3
0.2
0.1
0
Abs
250
300
350
400
450
Wavelength (nm)

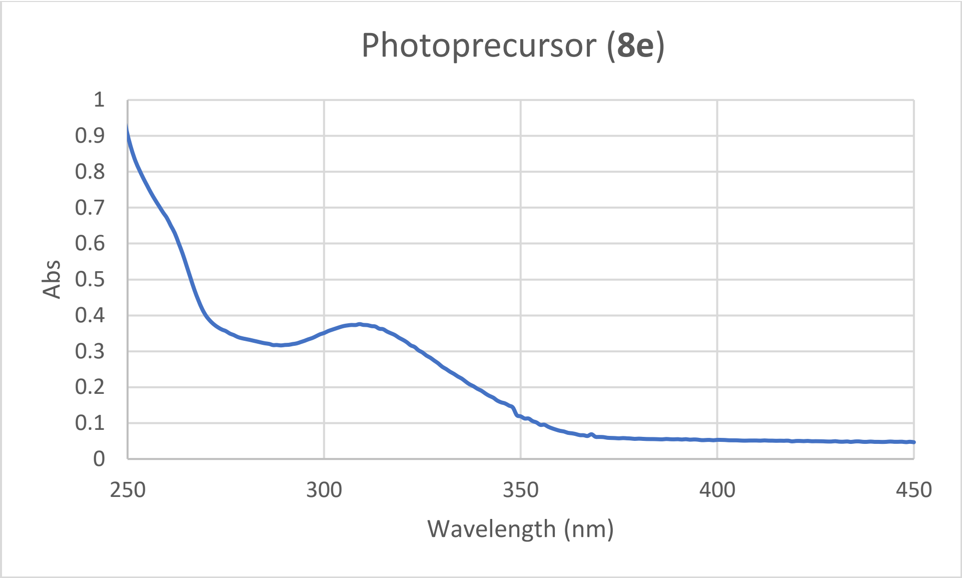
Photoprecursor (8e)
Abs
1
0.9
0.8
0.7
0.6
0.5
0.4
0.3
0.2
0.1
0
250
300
350
400
450
Wavelength (nm)

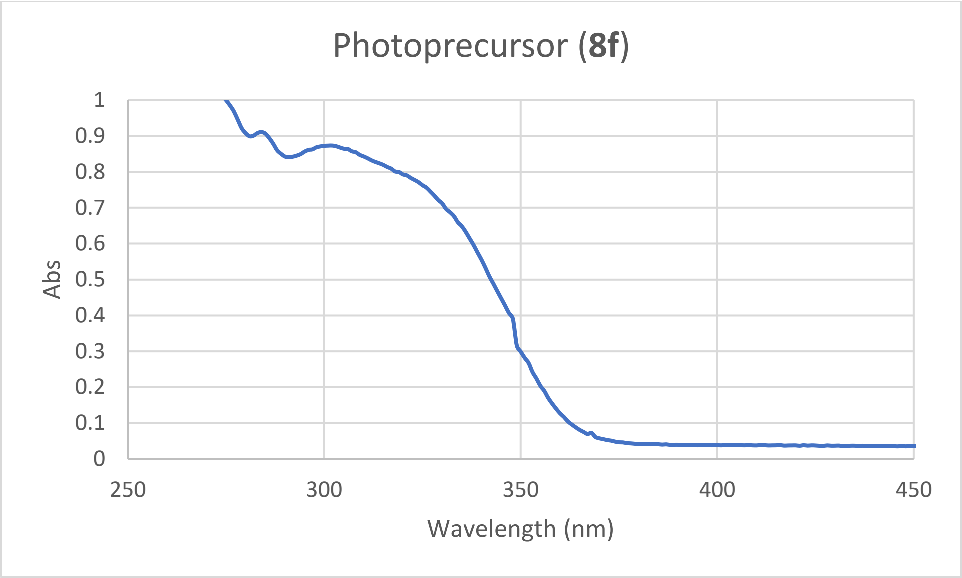

Photoprecursor (**8f**)
Abs
1
0.9
0.8
0.7
0.6
0.5
0.4
0.3
0.2
0.1
0
250
300
350
400
450
Wavelength (nm)

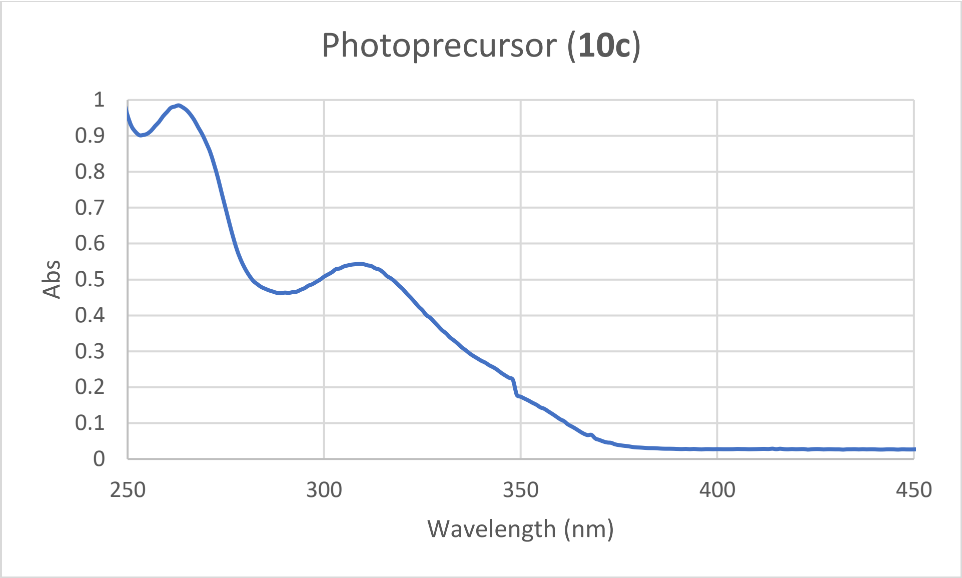
Photoprecursor (**10c**)
Abs
1
0.9
0.8
0.7
0.6
0.5
0.4
0.3
0.2
0.1
0
250
300
350
400
450
Wavelength (nm)
